# SCAPS simulation and design of highly efficient CuBi_2_O_4_-based thin-film solar cells (TFSCs) with hole and electron transport layers

**DOI:** 10.1038/s41598-025-12091-7

**Published:** 2025-08-03

**Authors:** Ahmed A. El-Naggar, Ahmed M. Eid, Yasmeen Rafat, Mohamed A. Khamis, Mabrouk Bakry, Salah Elkun, Walid Ismail, Swellam W. Sharshir, Abdelhamid El-Shaer, Mahmoud Abdelfatah

**Affiliations:** 1 Physics Department, Faculty of Science, Kafrelsheikh University, Kafrelsheikh, 33516 Egypt; 2 Nano Science and Technology Program, Faculty of Science, Kafrelsheikh University, Kafrelsheikh, 33516 Egypt; 3 Mechanical Engineering Department, Faculty of Engineering, Kafrelsheikh University, Kafrelsheikh, 33516 Egypt

**Keywords:** Simulation, Thin-film solar cell, Cu_2_O/CuBi_2_O_4_/TiO_2_, SCAPS-1D, Efficiency, Mathematics and computing, Physics

## Abstract

The continued rise in global temperatures and climate change has increased the demand for renewable energy sources. Recent developments in thin-layer photovoltaic cells have improved power output, affordability, and overall efficiency, spurred by the growing demand for renewable energy sources. In this study, numerical simulations of solar cells utilizing (SCAPS-1D) were employed to examine the efficiency of a CuBi_2_O_4_-based thin-film solar cell (TFSC). The CuBi_2_O_4_ absorber layer, known for its stability and optimal bandgap, was integrated with a Cu_2_O hole transport layer (HTL), CdS buffer layer, and TiO_2_ electron transference layer (ETL). Numerous constraints, including layer thickness, bandgap, and carrier concentration, were augmented to enhance the photovoltaic characteristics, such as fill factor (FF), open-circuit voltage (V_oc_), efficiency (η) and short-circuit current density (J_sc_). The study differentiates itself with a device structure constructed from Au/Cu_2_O/CuBi_2_O_4_/CdS/TiO_2_/FTO, which has impressive characteristics such as an open-circuit voltage of 1.2 V, a short-circuit current density of 32.85 mA/cm^2^, a fill factor of 88.42%, and an efficiency of 34.98% at lower defect density, although this efficiency exceeds the theoretical limit established by Shockley-Queisser limit for single-junction solar cells, it is essential to recognize that limit does not consider real-world constraints such as nonradiative recombination. The reported power conversion efficiency (PCE) of 32.56% was obtained under idealized simulation conditions, characterized by minimal bulk and interfacial defect densities. These findings not only affirm the promise of CuBi_2_O_4_ as an eco-friendly, low-cost absorber material but also underscore the importance of accounting for both intrinsic and extrinsic defect mechanisms in simulation-driven photovoltaic design.

## Introduction

The recent report reveals a 6.5% rise in global warming concerns^1^, necessitating swift progress in renewable power generation to meet the unprecedented demand^2^, and reducing CO_2_ emissions ranging between 400 and 1000 g per kilowatt-hour from fossil fuels^3^. Silicon solar panels emit zero CO_2_, potentially fulfilling about 15 TW of the world’s energy demands through solar energy alone^4,5^. First-generation crystalline silicon (c-Si) solar cells, which have an efficiency of 26.7%, dominate the global photovoltaic (PV) industry, accounting for 95% of the market^6,7^. Scientists address the complexities of experimenting with thin TFSCs with solar cell capacitance simulators (SCAPS). This simulator functions as a reliable 1D solar cell modeling software, enabling researchers to obtain results with remarkable specifications in terms of purity, accuracy, and thickness to optimize the utilization of these cells effectively ^8–10^.

Perovskites (PSCs) have significantly impacted third-generation PV technology in recent years^11^ due to their low raw material costs, high absorptivity, low atomic energy, simple production method, and they may emerge as a viable alternative to natural gas^12,13^. The perovskite structure denoted ABX_3_ has cations inhabiting sites A and B and anions, or halogens, occupying site X^14,15^. Moreover, CuBi_2_O_4_ cells have a higher Shockley-Queisser limit than perovskites because they have a photoelectron chemical stability in alkaline solutions, so interactions across visible light spectrums are possible and complex fabrication processes are overcome^16^.

A thin-film solar cell’s absorb layer plays a crucial role in its efficiency^17^. CuBi_2_O_4_ has emerged as a preferred absorber material owing to its identification as a p-type semiconductor with an optimal bandgap between 1.5 and 1.9 eV, providing a maximal photocurrent as high as 19.7–29.0 mA cm^-2^, and their conduction bands can be negatively charged beyond thermodynamic potential^18,19^. CuBi_2_O_4_ exhibits low human toxicity, an excessive optical absorption coefficient of greater than10^4^ cm^-1^, and a valence band with highly positive potential for facilitating electron transfer^20,21^. It also allows for substantial light penetration of approximately 280 nm at 550 nm while limiting charge-carrier diffusion to a wavelength range between 10 to 50 nm due to its restricted carrier movement ($\sim 1.2\times {10}^{-3}{\text{cm}}^{2}{\text{V}}^{-1}{\text{S}}^{-1})$. Maximizing TFSC power conversion efficiency involves implementing electron and hole transport layers, which facilitate charge separation, align interfacial energy levels, minimize charge recombination, and promote selective hole extraction and electron blocking^22,23^.

Inorganic HTLs outperform organic and polymeric ones in terms of long-term stability and cost-effectiveness in photovoltaic applications^24,25^. Cuprous oxide (Cu_2_O) is the preferred inorganic semiconductor for researchers as a result of its superior characteristics: holes with a mobility greater than 100 cm^2^/(V·s), extensive carrier diffusion length, and superior electrical properties^26,27^. Cu_2_O possesses a p-type direct bandgap of 2.07 eV^28^, and its elevated work function at the HTL interface results in an ohmic contact that minimizes open-circuit voltage (V_OC_) losses and production costs^29^. The combination of the CuBi_2_O_4_ solar structure’s rear minority electron mitigation and the Cu_2_O HTL resulted in a significantly improved efficiency compared with devices with other HTLs^30^. Compared to alternative HTL materials, Cu_2_O exhibited superior charge selectivity and contributed to a noticeable increase in both J_SC_ and overall efficiency^31,32^. Additionally, Cu_2_O offers a degree of environmental resilience, helping to reduce moisture-induced degradation^33^. Cu_2_O’s versatility across various photovoltaic device architectures is well documented. In heterojunction cells, it enhances charge separation when paired with complementary semiconductors. In homojunction designs, tuning the doping profile enables internal field generation for efficient operation. Its inclusion in tandem structures, particularly with crystalline silicon (c-Si), further extends spectral coverage and maximizes solar energy conversion^34^.

Electron transport layers (ETLs) significantly improve the efficiency of solar cells by suppressing trap states and managing ion migration, influencing hole transport significantly^35,36^. Titanium dioxide (TiO_2_) is a natural and low-toxic n-type material^37^ with superior thermal stability, chemical robustness, a simple fabrication process, remarkable photocatalytic properties, affordability, and suitable band alignment, with a bandgap between 3 and 3.2 eV. This feature promotes rapid electron transport and blocks holes at the junction^38^.

The buffer layer in a heterojunction plays a crucial function in establishing a large depleting region between it and the type p absorption barrier, hence optimizing light absorption by the latter. So it should have minimum absorption and surface recombination losses and low electrical impedance^39,40^. Cadmium sulfide (CdS) with n-type conductivity, high transparency, a direct energy gap transition between 2 to 2.4 eV, and an efficient electron affinity of 4.2 eV is a buffer layer, extending the carrier lifetime and optimizing band alignment^41,42^. Although CdS remains a subject of environmental concern due to the presence of cadmium, its widespread use persists owing to its nearly ideal conduction band alignment with various absorbers and its excellent crystallographic compatibility. The decision to use CdS in our study was based on its proven performance, with the understanding that future work may explore cadmium-free alternatives. Furthermore, we stress the necessity of proper recycling protocols to minimize environmental risks associated with Cd-containing devices^43^. New back-contact structures were designed to prevent transmission loss under illumination^44^. There are several types of back contacts available on the market, but gold is a better choice as it is a more stable and conductor of electricity^45^. Gold (Au) was selected as the rear contact material based on its excellent work function and well-established compatibility with copper-based absorber layers, which together promote favorable band alignment and efficient hole extraction. Although molybdenum (Mo) is a widely adopted and more cost-effective alternative, our simulations showed that Au provided marginally better performance in terms of both efficiency and carrier selectivity^46^.

While CuBi_2_O_4_ has been explored as an absorber material in previous studies, this work presents a unique integration of Cu₂O as a hole transport layer (HTL) with CuBi_2_O_4_ as the absorber, alongside CdS as a buffer layer and TiO_2_ as an electron transport layer (ETL). This specific device architecture, consisting of Au/Cu_2_O/CuBi_2_O_4_/CdS/TiO_2_/FTO, leads to impressive photovoltaic characteristics, including an open-circuit voltage (V_oc_) of 1.2 V, a short-circuit current density (J_sc_) of 32.85 mA/cm^2^, and a power conversion efficiency (PCE) of 32.56%. These values are notable and demonstrate the potential of this novel’s configuration. Furthermore, the study optimizes key parameters such as layer thickness, bandgap, carrier concentration, and defect density, showing how these factors collectively enhance the performance of CuBi_2_O_4_-based thin-film solar cells. While the Shockley-Queisser limit for single-junction solar cells sets theoretical boundaries, this design approach accounts for real-world factors like non-radiative recombination, which allows for a deeper understanding of practical device performance.

## Apparatus’s construction and simulation methodology

**Fig. 1:**
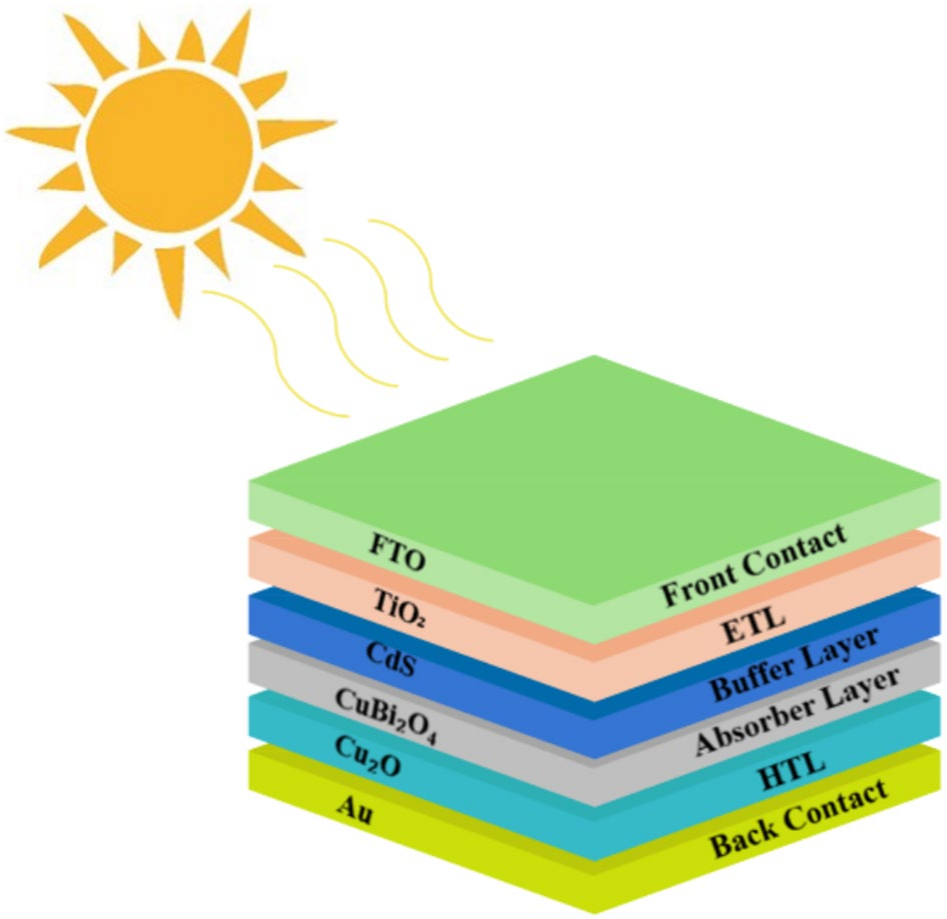
An illustration of a proposed solar cell schematic (Au/Cu_2_O/CuBi_2_O_4_/CdS/TiO_2_/FTO).

The device’s construction is depicted in Fig. 1, where the Au/Cu_2_O/CuBi_2_O_4_/CdS/TiO_2_/FTO sequencing is shown. It consists of the following components: A front contact made of FTO, a hole transport layer made of Cu_2_O, a buffer layer made of CdS, an absorb layer made of CuBi_2_O_4_, a novel electron transport layer made of TiO_2_, and a back contact made of Au. At the CuBi_2_O_4_/CdS and Cu_2_O/CuBi_2_O_4_ layers, interface defects are introduced to the structure of the layers. As part of the investigation, each layer of the structure will be subjected to a series of experiments involving variables such as thickness, band gap, carrier concentration, defect density, and temperature. By employing SCAPS-1D, the short circuit current (Jsc), efficiency (η), open circuit voltage (V_oc_), and fill factor (FF) will be analyzed for each parameter.

### Material parameters and computational modeling

During this study, SCAPS-1D software (Version 3.3.10) was utilized to model and analyze the performance of TFSCs^47^. It can allow researchers to simulate various physical processes in solar cells (SCs)^48^. Other programs, including the Advanced Modeling Program for Semiconductors 1D (AMPS-1D) and the Advanced Modeling Program for Solar Cells (wxAMPS)^49^, are used to model and simulate semiconductor devices and analyze solar cell performance^50^. Each program has its own unique features that can be the key difference between one program and another^37,51^. Using accurately assessed simulation settings for several layers, the SCAPS-1D simulator enables the creation of many junctions, heterojunction, and homojunction SCs architectures^52^. Being created especially to model and simulate TFSC, SCAPS-1D is the most efficient program. It includes complex simulations of carrier generation, recombination, transport, and interface effects in the layers of TFSC^53^. Additionally, it enables the modeling of flaws and interface features among the layers of the SC^54^. This competence is essential to comprehend and maximize the reliability and effectiveness of devices. Another intriguing aspect of SCAPS-1D is how quickly it simulates, which is effective because other programs may lag considerably^55^. Thalso provides the most precise numerical results with a straightforward interface. The J-V curve, frequency capacitance (C-F), loss of charge carriers (recombination), external quantum efficiency (EQE), illustration of an energy band diagram, cell resistances, and working temperatures are a few of the outputs that may be acquired using the SCAPS 1D software^3,26^. We thoroughly examined how the thickness, bandgap, electron and hole mobilities, electron affinity, and concentration of carriers of the absorbent and window layers affect basic parameters essential to SC’s efficiency and effectiveness. A variety of inputs were used to identify which of the above parameters would best improve the efficiency of SC devices. The program combines several studies to offer an in-depth understanding of SCs performance and behavior by providing precise outputs^56^. An extensive list of the elementary parameters for the materials is provided in (Table 1). Detailed information about the interface and contact parameters can be found in (Tables 2, 3).

**Table 1:** Describes the simulation parameters.

Materials’ characteristics	Cu_2_O	CuBi_2_O_4_	CdS	TiO_2_
Thickness (nm)	400	3000	60	50
Dielectric permittivity	7.1	34	9	10
Bandgap (eV)	2.2	1.40	2.4	3.2
Affinity of electrons (eV)	3.2	3.72	4	4.2
Density of valence band maximum states (cm^−3^)	1.1 × 10^19^	5 × 10^19^	1.8 × 10^19^	2 × 10^17^
Conduction band minimum density (cm^−3^)	2.1 × 10^17^	1.2 × 10^19^	2.2 × 10^18^	6 × 10^17^
Concentration of acceptors (cm^-3^)	0	8 × 10^19^	1 × 10^18^	0
Concentration of donors (cm^−3^)	1 × 10^19^	0	0	1 × 10^17^
Mobility of electron (cm^2^/(V s))	2 × 10^2^	1.1 × 10^–3^	1 × 10^2^	1 × 10^2^
Mobility of hole (cm^2^/(V s))	8 × 10^1^	1.2 × 10^–3^	2.5 × 10^1^	2.5 × 10^2^
Velocity of the electron thermally (cm/s)	10^7^	10^7^	10^7^	10^7^
A hole’s thermal velocity (cm/s)	10^7^	10^7^	10^7^	10^7^

**Table 2:** Presents the interface parameters.

Interface parameter	Cu_2_O/CuBi_2_O_4_	CuBi_2_O_4_/CdS
Type of defect	Neutral	Neutral
Capture cross section of hole (cm^2^)	10^–19^	10^–19^
Capture cross section of electron (cm^2^)	10^–19^	10^–19^
Energy distribution	Single	Single
Nt (cm^2^)	10^7^–10^18^	10^7^–10^17^
Referential energy (eV)	0.6	0.6

**Table 3:** The contact parameters of the simulation^57,58^.

Contacts	Back metal contact properties (Au)	Back metal contact properties (Mo)	Front metal contact properties (FTO)
Function of metal work (eV)	4.98	4.95	4.07
Speed of electron recombination at the surface (cm/s)	1.000 × 10^7^	1.000 × 10^5^	1.000 × 10^7^
Amount of surface recombination in a hole (cm/s)	1.000 × 10^7^	1.000 × 10^7^	1.000 × 10^7^

The SCAPS-1D uses continuity equations , Poisson’s equation, and carrier transport to assess TFSC^31^, which can be obtained in the following Eq. (1,2):

1 $$\frac{{d}^{2}}{{dx}^{2}}\varphi \left(x\right)=\frac{e}{{\varepsilon }_{0}{\varepsilon }_{r}}(p\left(x\right)-n\left(x\right)+{N}_{D}-{N}_{A}+{\rho }_{defect}\left(p\left(x\right), n\left(x\right)\right))$$

2 $$\frac{d{J}_{n}}{dx}=G-R and \frac{d{J}_{P}}{dx}=G-R$$

where the variables observed in the equations are potential of electrostatic (Ψ), electron charge (e), permittivity of relative (ε_r_), permittivity of vacuum (ε_0_), electron distribution (ρ_n_), electron density (n), hole density (p), hole distribution (ρ_p_), current densities of electrons (J_n_), hole current densities (J_p_), rate of recombination (R), and generation rate (G), they are denoted^31,59^. Furthermore, certain properties, hole and electron thermal velocities, both 10^7^ cm/s, remain constant throughout all layers.

## Results and discussions

Simulations of an inorganic TFSC were carried out using the software. During the simulation, the base temperature is 300 K, the illumination is AM1.5G, and the photon intensity is 100mW/cm^2^^60^.

### Performance of solar cells with HTL materials

The higher conductivity of HTLs significantly influences J_sc_ in conventional TFSCs^61^. Compared with other HTLs, the proposed device using Cu_2_O exhibits higher J_sc_ and PCE^62^.

#### Impact of Cu_2_O Thickness on bandgap variation

**Fig. 2:**
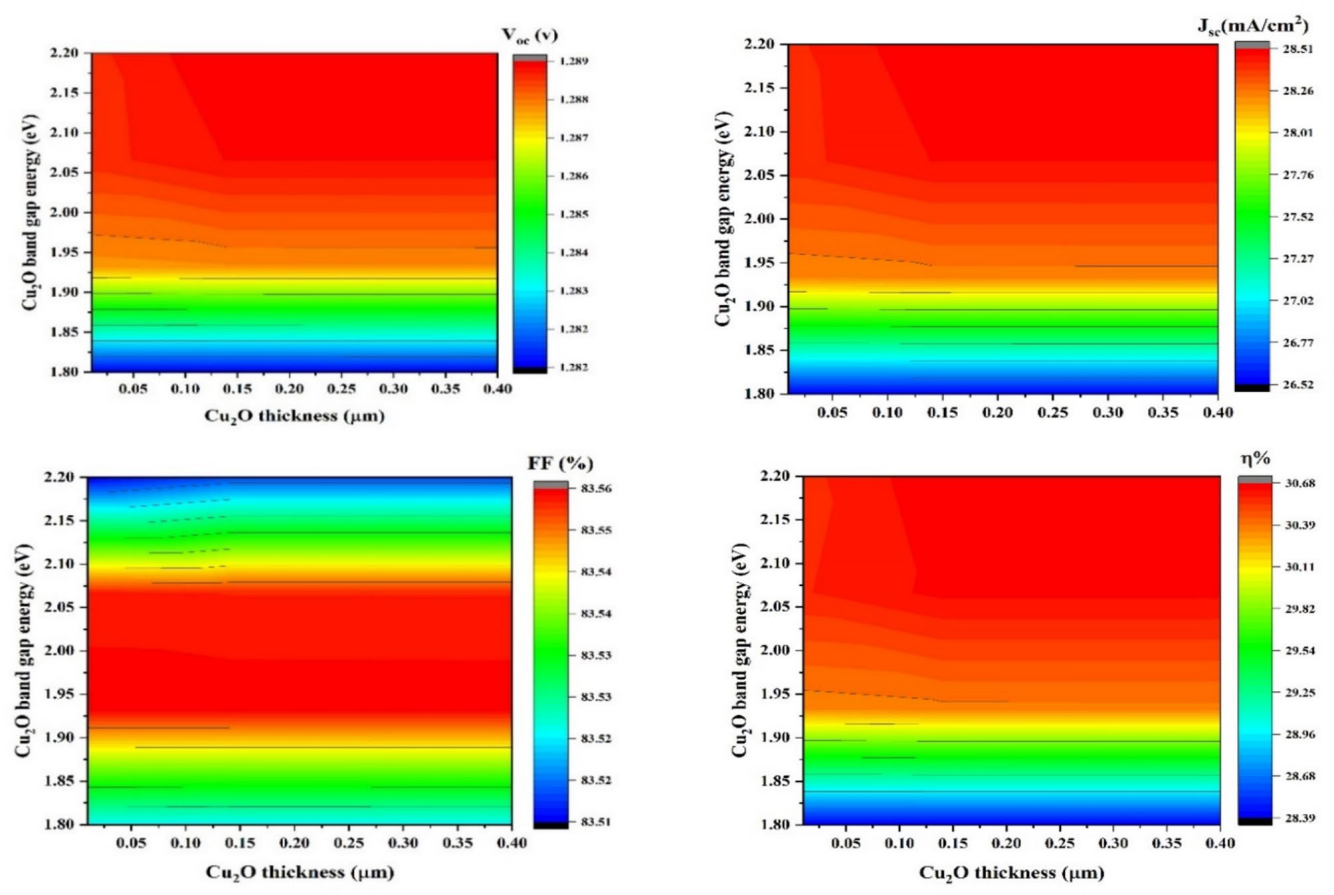
Effect of the thickness(x-axis) vs band gap (y-axis) of Cu_2_O on (**a**) V_oc_ , J_sc_ (**b**), (**c**) FF_,_ and (**d**) η_._

The HTL thickness should be carefully determined to ensure complete coverage of the absorber layer and to maintain a sufficiently thin profile to minimize recombination^63^. The HTL with a higher HOMO level efficiently extracted holes in Cu_2_O until the band gap reaches 2.24 eV^64,65^. FF and PCE increased with HTM up to 0.02 µm. Above 0.05 µm, we noticed a constant value for V_oc_, FF, J_sc_, and PCE, as shown in Fig. 2, which means the thickness that gives optimum performance ranges from 0.04 to 0.16 µm. According to Fig. 2a, V_oc_ improved from 1.286 near 1.289 v through an growing bandgap from 1.9 to 2.20 eV. Figure 2b, J_sc_ increased from 27.76 to 28.51 mA/cm^2^ with a rising band gap from 1.9 to 2.20 eV. Figure 2c, *FF* increased from 83.54 to 83.56% with an increasing bandgap from 1.95 to 2.10 eV but decreased up to 2.10 or less 1.9 eV, as the HOMO level of Cu_2_O must exceed the valence band to boost the hole extraction efficiency and Fig. 2d, η increased from 29.82% to 30.68% through an increasing gap of energy from 1.9 to 2.20 eV but decreased with an energy gap smaller than 1.9 eV results in enhanced light absorption and decreased electrical conductivity.

#### Impact of Cu_2_O thickness on carrier concentration variation

**Fig. 3:**
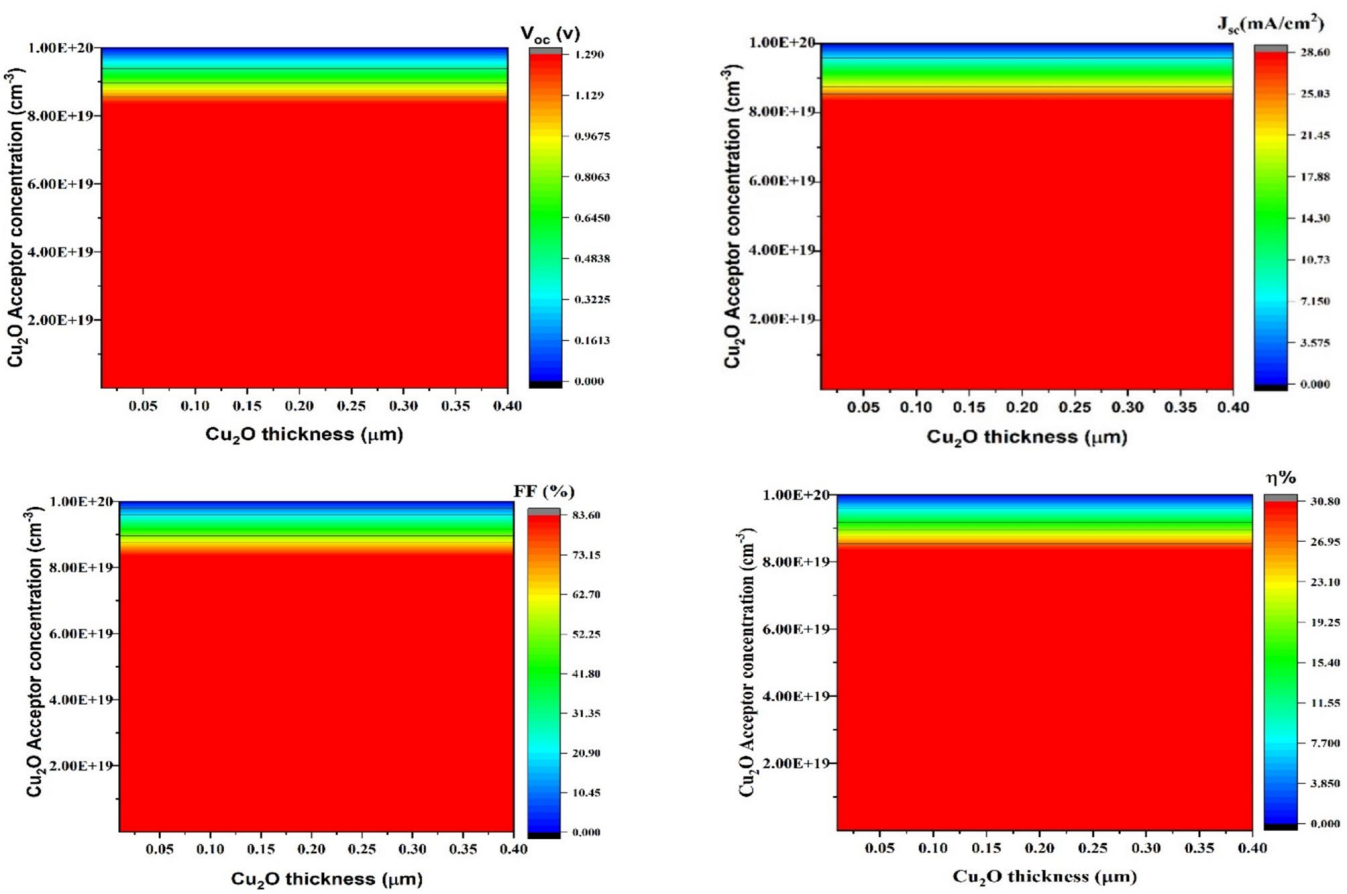
Effect of the carrier concentration (y-axis) of Cu_2_O thickness (x-axis) on (**a**) V_oc_, J_sc_ (**b**), (**c**) FF_,_ and (**d**) η_._

Boosting the charge carrier density in the HTL can improve the overall efficiency of TFSC cell extraction and collection^66^. A higher HOMO energy level in the hole transport material (HTM) improves the charge transport efficiency, minimizes recombination, and decreases series resistance, thereby boosting SCs performance^67,68^. For Cu_2_O HTL carrier concentrations exceeding 10^18^ cm^-3^, the PCE increased. A high doping density at the Cu_2_O/CuBi_2_O_4_ contact leads to a substantial built-in voltage, causing low carrier recombination loss^69^. As doping concentration increased, the Fermi level moved nearer to the edge of valence band^70^. Figure 3 Demonstrates SCs- PV performance parameter changes according to varying Cu_2_O HTL carrier concentrations. As the Cu_2_O HTL carrier concentration surpasses 10^18^ cm-^3^, the four PV performance parameters display a decreased recombination of photogenerated charges and enhanced electrical conductivity. Hence, 10^18^ cm^-3^ is the best concentration of carriers for Cu_2_O HTL. Figure 3a,b V_oc_ and J_sc_ increase from 1.129 to 1.290 V and from 25.03 to 28.6 mA/cm^2^, respectively, with carrier concentration 10^18^ cm^-3^–10^19^ cm^-3^, (c) FF increases since 73.15 to 83.60% with carrier concentration > 10^18^ cm^-3^ but decreases with carrier concentration > 10^19^ cm^-3^ due to decreasing electrical conductivity, and (d) shows an increase of *η* from 26.95 to 30.80% with decreasing carrier concentration < 10^20^ cm^-3^.

#### Impact of Cu_2_O thickness on J-V curve

**Fig. 4:**
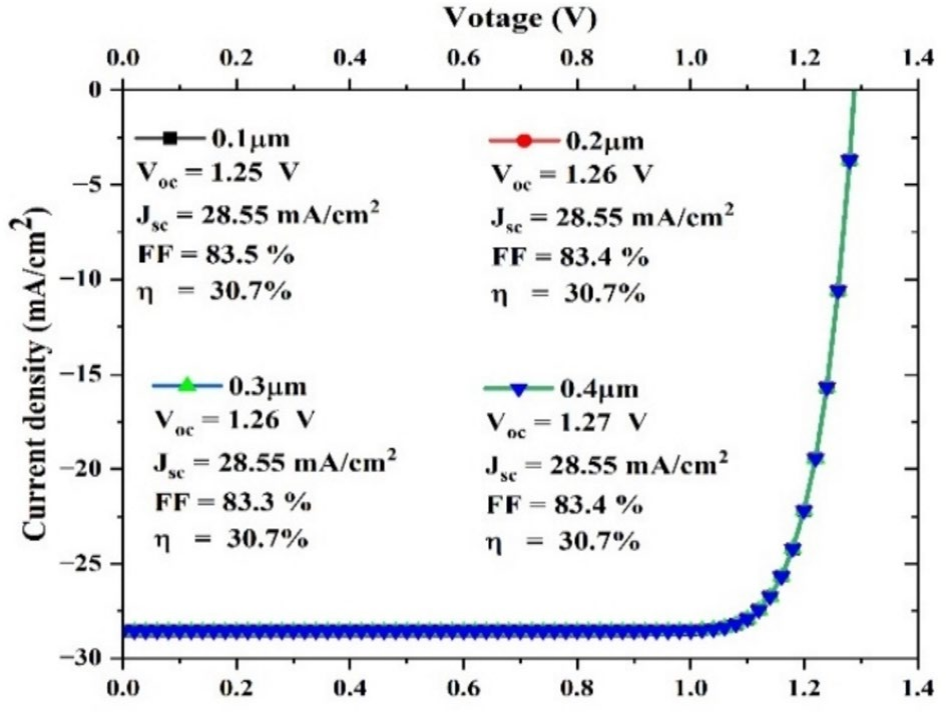
J-V curve of Cu_2_O HTL by different thicknesses.

The J-V features in Fig. 4, illustrate the variation in performance between 0.1 and 0.4mm Cu_2_O absorb layers. A linear fit to the experimental data was used to determine the Cu_2_O films’ sheet resistance RSH. With increasing HTL (Cu_2_O) thickness, photovoltaic output remains constant. SC’s HTL is subjected to sunlight only after traversing the active absorber layer. As a result of the absorb and buffer layers absorbing a majority of the photons, the HTL is only left with a minimal amount of photons to absorb. The alterations in the HTL thickness did not affect the number of photogenerated charge carriers. The SCs performance is not affected. Short-circuit current is modestly increased as voltage increases due to the decrease in Cu_2_O work function, which contributes to the rise in short-circuit current^71^. The modification of photovoltaic characteristics such as V_OC_, FF, J_SC_, and efficiency as a result of varying Cu_2_O thickness is depicted in (Fig. 4). Optimal thickness of 0.1 µm, FF and *η* have a maximum value, but V_oc_ has a minimum value, and J_sc_ is constant.

#### Impact of Cu_2_O thickness, carrier concentration, and band gap on EQE

**Fig. 5:**
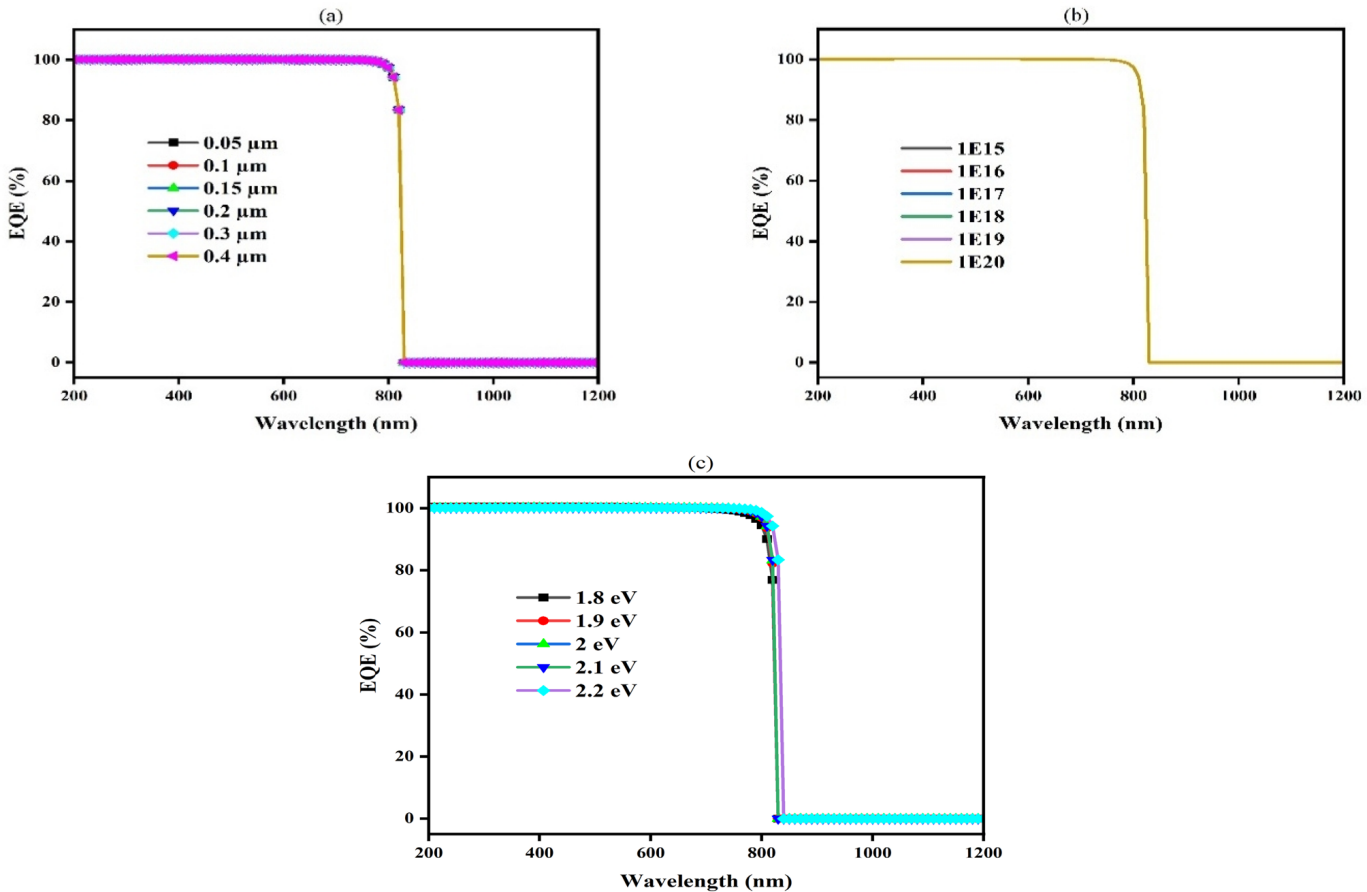
Influence of thickness (**a**), donor concentration (**b**), and (**c**) band gap on EQE of Cu_2_O.

The EQE of CuBi_2_O_4_ TFSC through Cu_2_O HTL occurs between 400 and 800 nm wavelengths. There is a possibility that the absorber has removed sufficient charge carriers. From Fig. 5, the EQE of the Cu_2_O layer is calculated according to wavelength with the difference of thickness Fig. 5a, donor concentration (Fig. 5b), and energy gap of the layer as illustrated in (Fig. 5c). EQE is observed to have the same shape and value for different values of thickness and donor concentration, with a value of 100% at a wavelength from 200 to 830 nm. After this wavelength, the photon light cannot be absorbed. And it was observed that the EQE is also 100% with different values of band gap, but the maximum absorbed wavelength increases from 830 to 840 nm with a 2.2 eV band gap.

### Buffer material impact on solar cell performance

Variations in CdS layer thickness, donor concentration, and band gap as well as its influences on J_sc_, V_oc_, and FF with the remaining restrictions of the other layers fixed.

#### Impact of CdS thickness on band gap variation

**Fig. 6:**
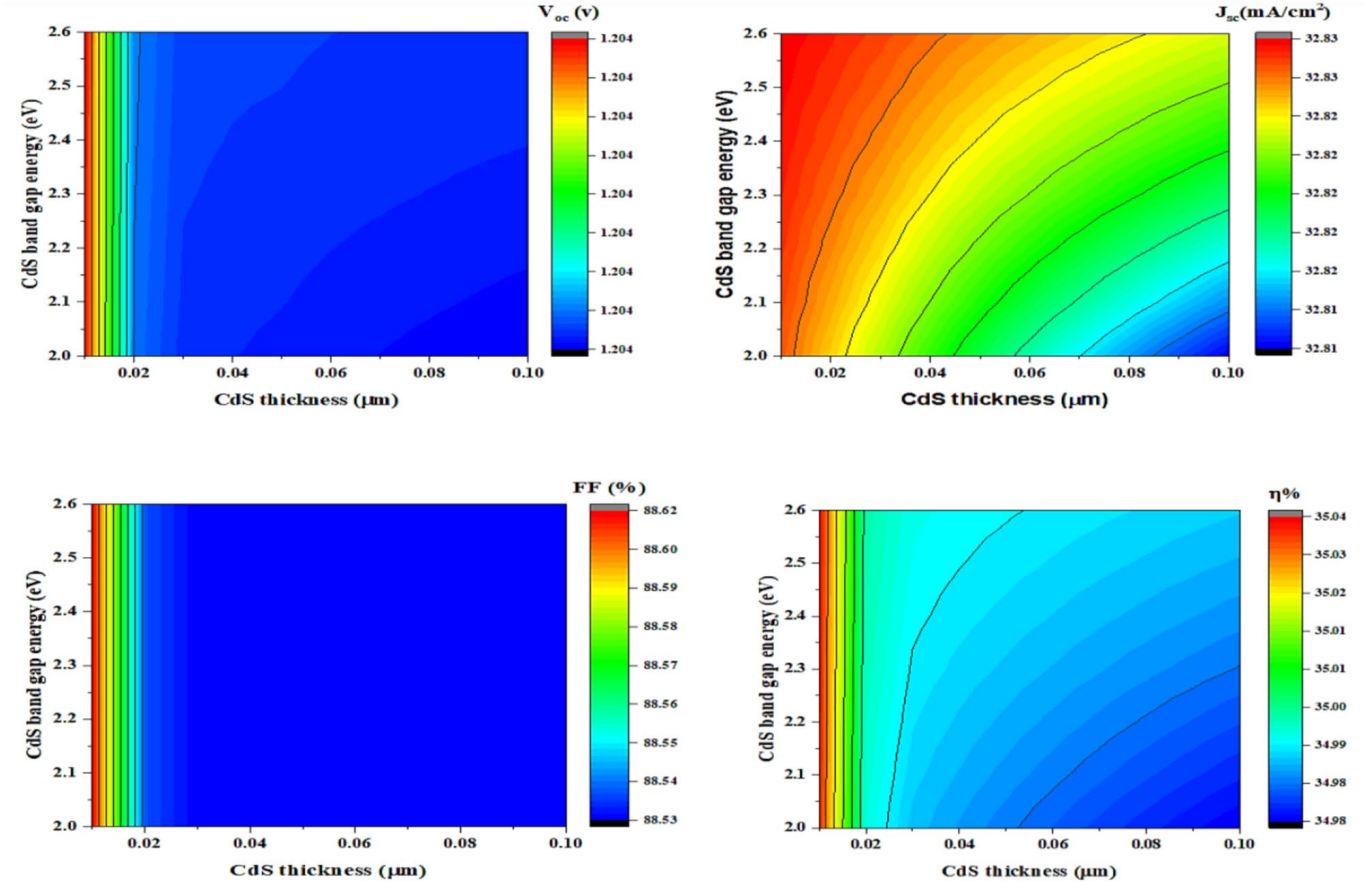
Effect of thickness on x-axis and band gap on y-axis of CdS on (**a**) V_oc_, (**b**) J_sc_, (**c**) FF, and (**d**) η.

Figure 6 calculates J_sc_, V_oc_, η, and FF according to the difference in the band gap and thickness of CdS from 2.0 to 2.6 eV (y-axis) and from 0.01 to 0.10 µm (x-axis), respectively. Figure 6a, explains that V_oc_ decreases very slightly from 1.2043 to 1.2041 V by approximately 0.0002 at a thickness between 0.01 and 0.02 µm, and then the depth since 0.02 near 0.10 µm is almost constant at 1.2041 V. As the CuBi_2_O_4_ layer generates charge carriers, the inherent potential gradient that helps in its separation and extraction diminishes, thus increasing the recombination rate. The V_oc_ remains constant as the energy gap rises from 2.0 to 2.6 eV. More effective electron separation can be facilitated when the edge band conduction of CdS approaches or better aligns with the conduction band of CuBi_2_O_4_, leading to rather stable V_oc_ or possibly even modest enhancement if recombination losses are decreased. Figure 6b, shows that J_sc_ rises from 32.81 to 32.83 mA/cm^2^ when the energy gap increases up to 2.6 eV, as a wider band gap of CdS may result in better light transmission from the CdS layer to the CuBi_2_O_4_ absorber layer, absorbing more photons and boosting J_sc_ in the process. The J_sc_ decreases between 32.83 and 32.81 mA/cm^2^ with a rising thickness from 0.01 to 0.10 µm on account of increased light absorption, which would boundary the quantity of light that reaches the CuBi_2_O_4_ layer absorber with a high thickness^70^. Figure 6c, clarifies that FF has nearly similar behaviour to the V_oc_ in Fig. 6a, as FF is still constant when the band gap increases with a value from 2.0 to 2.6 eV. Although certain parameters may change, such as a decrease in series resistance due to a better material quality, if the CdS band gap is increased, the direct effect on FF might not be as great as it is for J_sc_. It decreases from 88.62 to 88.53% when increases the thickness since 0.01 to 0.02 µm and then remains constant with thickness from 0.02 to 0.10 µm due to the possibility that it will raise the cells’ internal series resistance, which lowers the FF^71^. Figure 6d, exhibits that η decreases from 35.4 to 34.98% with an enhancement in the thickness with values from 0.01 to 0.10 µm. The η stays constant when the band gap rises from 2.0 to 2.6 eV increasing the thickness from 0.01 to 0.025 m and then increases from 0.025 to 0.10 µm.

#### Impact of CdS thickness on donor concentration variation

**Fig. 7:**
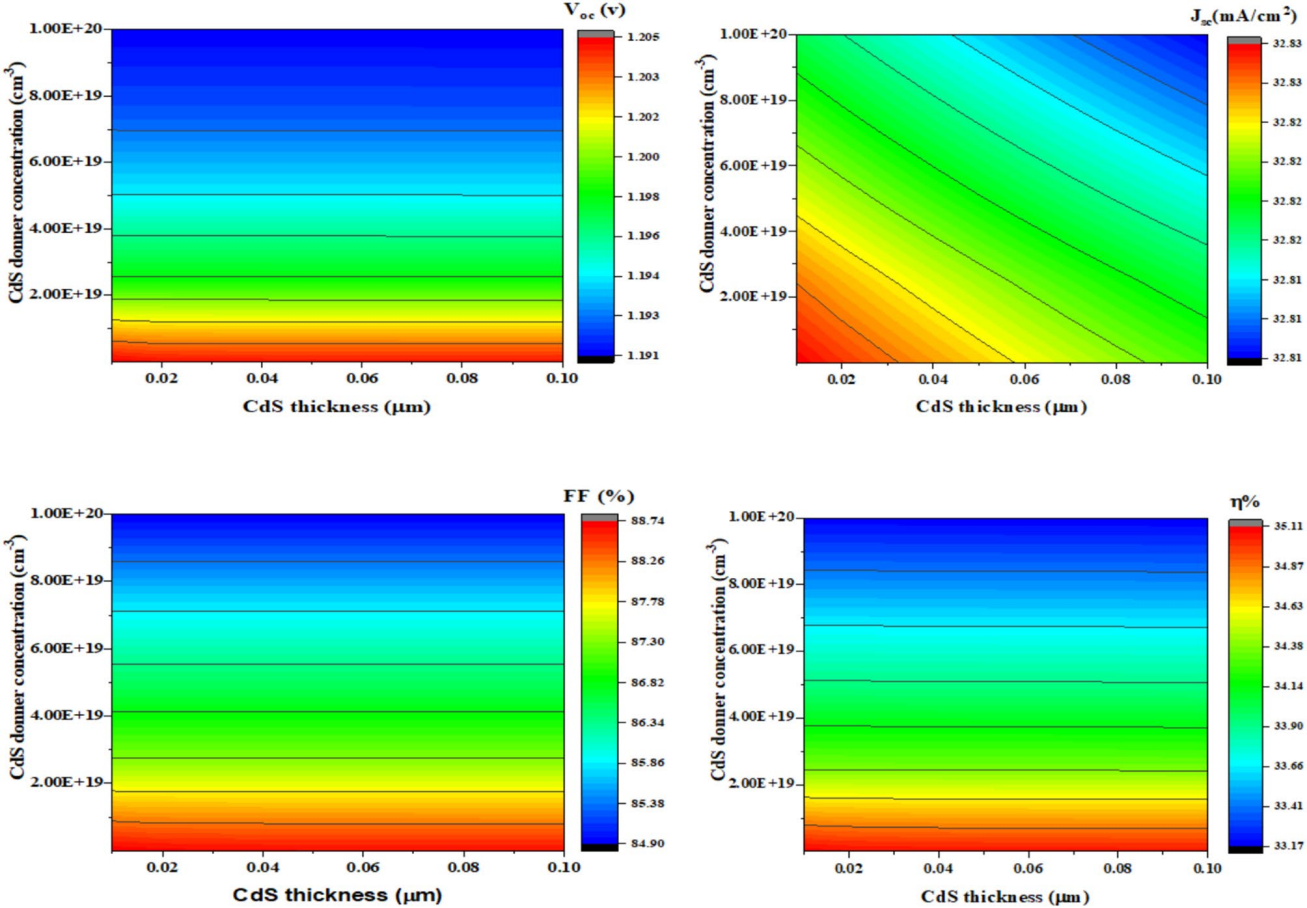
Effect of thickness (x-axis) and donor concentration (y-axis) of CdS on (**a**) V_oc_, (**b**) J_sc_, (**c**) FF and (**d**) η.

Figure 7 calculates J_sc_, V_oc_, η, and FF according to the difference in the donor concentration and thickness of CdS since 10^16^ to 10^20^ cm^-3^ (y-axis) and since 0.01 to 0.10 µm (x-axis), respectively. Figure 7a, provides that V_oc_ stays constant with the rise in thickness from 0.01 to 0.10 µm^37^. While the V_oc_ decrease since 1.205 near 1.191 V with the growing donor concentration since 10^16^ to 10^20^ cm^-3^ due to the increasing defects, the recombination process increases^70^. We studied the change in J_sc_ in (Fig. 7b), which turned out to be reduced from 32.83 to 32.81 mA/cm^2^ when the width rose from 0.01 to 0.10 µm. And the J_sc_ decreased from 32.83 to 32.81 mA/cm^2^ as the donor concentration rose from 10^16^ to 10^20^ cm^-3^ because of changes in light absorption and increase recombination.

Figure 7c indicates that FF keeps constant during the rise of thickness from 0.01 to 0.10 µm, and the internal parasitic resistance and band offset, and b determine FF and Voc. Both are not obstructed through the buffer layer thickness^37^. The FF decreased from 88.74 to 84.90% as the donor concentration rose from 10^16^ to 10^20^ cm^-3^. We calculate the η in (Fig. 7d), which demonstrates that η is still fixed when the thickness rises from 0.01 to 0.10 µm, but the η reduces from 35.11 to 33.17% at the donor concentration, which varies from 10^16^ to 10^20^ cm^-3^ depending on the previous results.

#### Influence thickness of CdS happening J-V curve

**Fig. 8:**
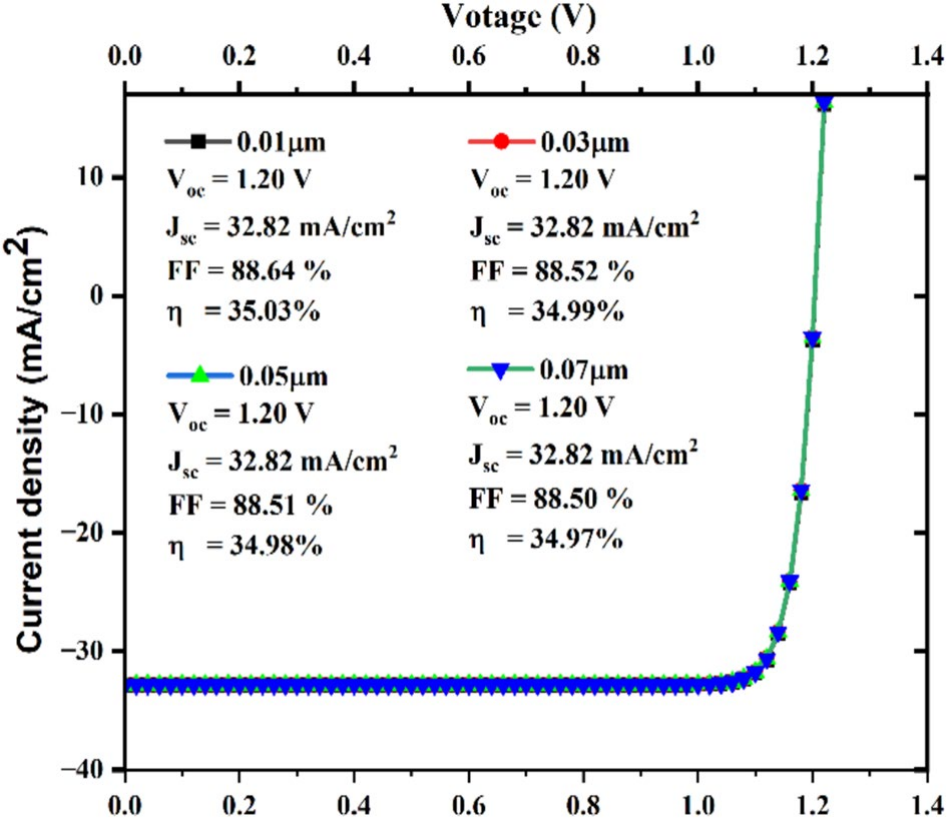
JV curve of solar cell with varying of CdS buffer layer thickness from 0.01 to 0.07 µm.

It has been found that a band gap of 2.45 eV is preferable, and the CdS thickness varies between 0.01 and 0.07 µm. The JV curve was plotted on (Fig. 8). On behalf of V_oc_, J_sc_, η, and FF of the SCs according to the thickness of the CdS buffer layer, which varieties since 0.01 to 0.07 µm at a 2.45 eV band gap. Figure 8, shows that the V_oc_ is constant, J_sc_ is constant, FF reduces from 88.64 to 88.50%, and η decreases from 35.03 to 34.97% with increasing thickness.

#### Measurement of EQE with different CdS thicknesses, carrier concentrations, and band gaps

**Fig. 9:**
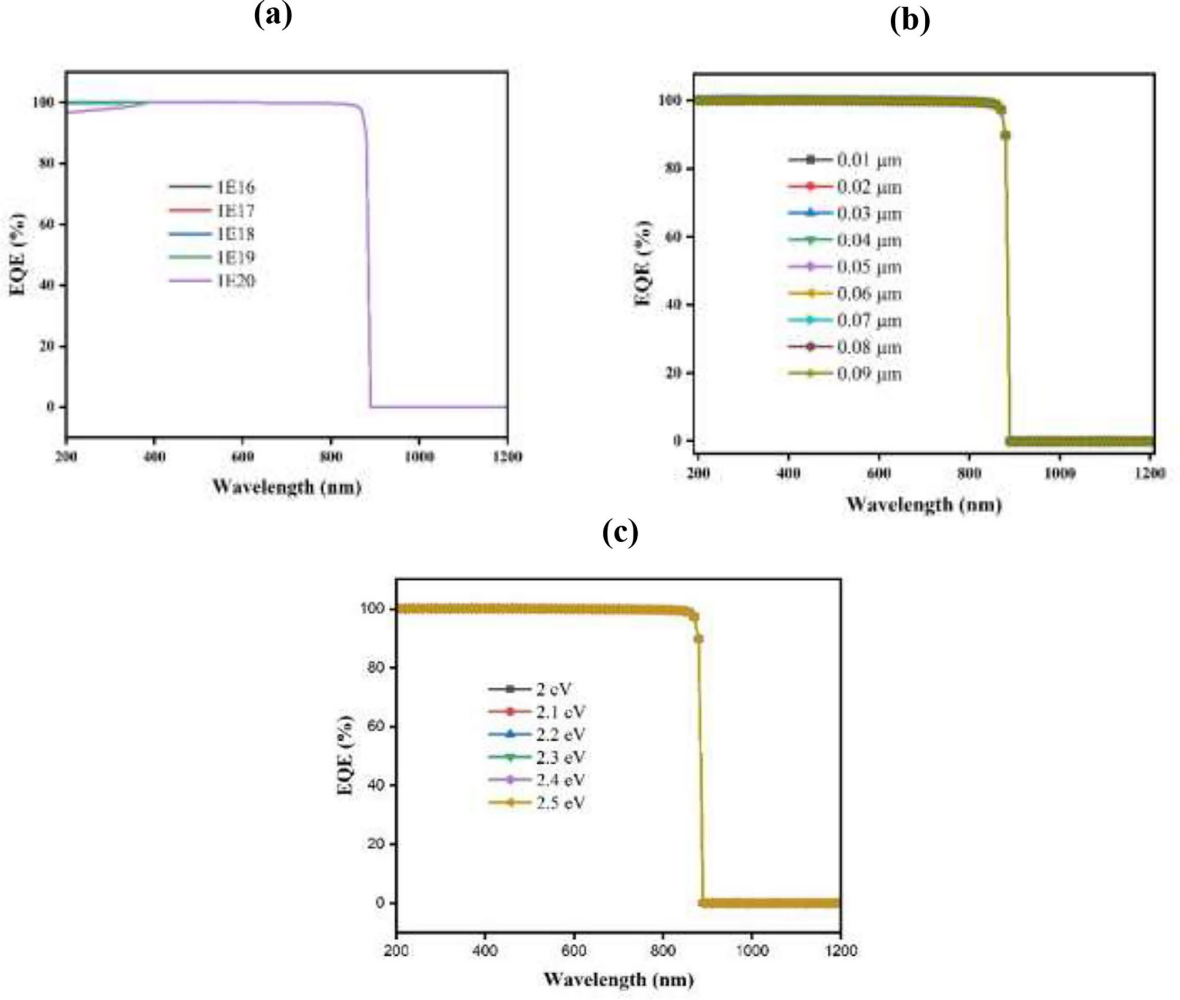
Influences of (**a**) thickness, (**b**) donor concentration, and (**c**) band gap on EQE of CdS buffer layer.

The EQE of the CdS buffer layer is calculated according to wavelength with the difference values of thickness from 0.01 to 0.09 µm as shown in (Fig. 9a), donor concentration from 10^16^ to 10^20^ cm^-3^ (Fig. 9b), and band gap between 2 and 2.5 eV (Fig. 9c). EQE is observed to have the same shape and value for the different values of thickness and band gap, with a value of 100% at wavelengths from 200 to 890 nm. The donor concentration has the same value of EQE for values from 10^16^ to 10^19^ cm^-3^ at 100% as shown, but at 10^20^ cm^-3^ it starts with a value of 96% and increases until it reaches 100% from wavelength 200 to 390 nm and then remains constant from 390 to 890 nm. After this wavelength, the photon light cannot be absorbed.

### Impact of absorption material on the performance of solar cells

The absorber layer is an important component of SCs that influences the model’s efficiency and performance. SCs are based on the photovoltaic effect, which converts sunlight into electricity. The absorber layer is at the focal point of this process, as it absorbs sunlight and produces charge carriers (holes and electrons). Factors such as absorb layer thickness, band gap, and material quality can affect the efficiency of the absorb layer.

#### Impact of CuBi_2_O_4_ thickness on band gap variation

**Fig. 10:**
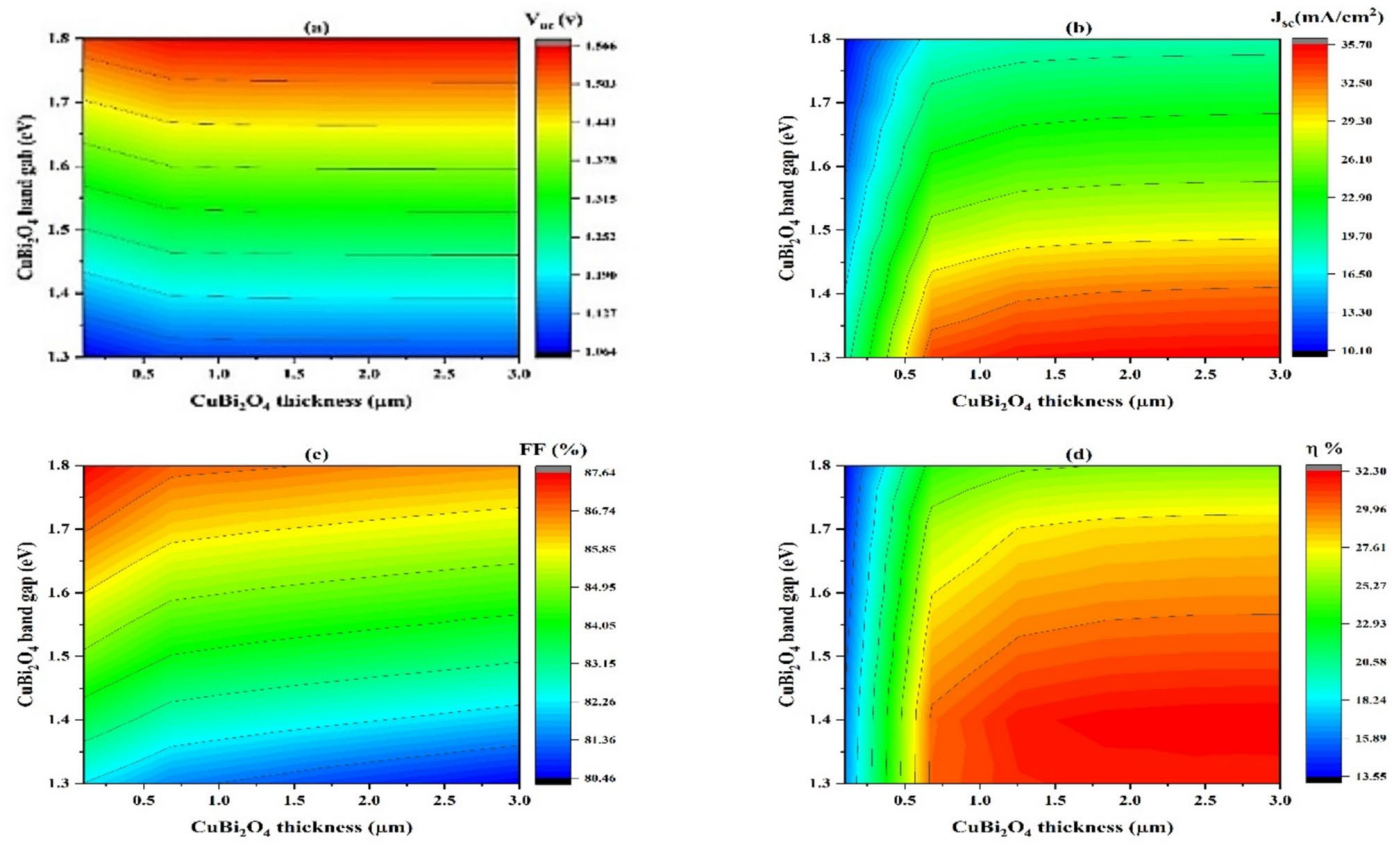
Key features of the modeled SCs, displaying the open-circuit voltage (V_oc_) (**a**), short- current circuit density (J_sc_) (**b**), fill factor (FF) (**c**), and (**d**) efficiency (η) in relation to the thickness absorber layer (x-axis) and band gap (y-axis).

For SCs to be effective, the absorb layer’s thickness and band gap must be adjusted^72,73^. Based on the simulation of different absorb layer thicknesses and band gaps, V_oc_, J_sc_, FF, and η at each given value were calculated. Figure 10 presents a contour plot illustrating the relationship between three variables: On the horizontal axis, CuBi_2_O_4_ ranges in thickness from 0.5 to 3.0 m.; The CuBi_2_O_4_ band gap, depicted on the vertical axis, varies between 1.3 to 1.8 eV; and Jsc, Voc, FF, and efficacy which represent the color gradient. The red color, and lower values by the blue color indicate higher values.

According to (Fig. 10a), the V_oc_ changes with variation in CuBi_2_O_4_ thickness and energy gap. V_oc_ growths since 1.064 V to 1.566 V as the energy gap and thickness of the absorber layer increase. The maximum V_oc_ (1.566 V) at a higher band gap approximately ranges from 1.75 to 1.8 eV, with a higher layer absorber thickness (3 µm). This may well described by the fact that thicker absorber layers are more effective at capturing and absorbing incoming photons, particularly in materials with high absorbing coefficients. This enhanced absorption causes a higher rate of production of electron–hole pairs^74^. The increase in CuBi_2_O_4_ absorber thickness was found to enhance the absorption of high-energy (short-wavelength) photons, leading to a higher generation of photocarriers. This, in turn, improves the short-circuit current density (J_SC_) and contributes positively to overall device performance. As a result, diminished recombination losses, higher photon absorption, raised built-in potential, and efficient carrier transit and collection all contribute to the increase in V_oc_.

As demonstrated by Fig. 10b, the short-circuit current density (J_sc_) increases roughly from 10.10 to 35.7 mA/cm^2^ as the band gap decreases since 1.8 to 1.3 eV and the thickness of the absorber layer ranges from 0.6 to 3 µm. Lower band gaps (1.3—1.4 eV) and thicker layers (2.5—3 µm) result in the highest J_sc._ (35.70 mA/cm^2^). Also, the lowest J_sc_ values are found where the CuBi_2_O_4_ thickness is lowest, and the bandgap is highest. Lower band gap and a thicker absorber layer can reduce band-to-band recombination and surface recombination, where holes recombine and electrons directly. This can donate to a higher J_sc_^75^.

In Fig. 10c, it is found that FF is reduced to increase further thickness, and energy gap of the layer absorber. FF increases with energy gap but has a more complex reliance on thickness. FF values rise from 86.74% and become higher at 87.64% at a lower thickness of the absorber layer (lower than 0.5 µm) and an energy gap greater than 1.7 eV. This can be due to materials with larger band gaps having fewer thermally produced carriers, resulting in lower inherent recombination rates. Furthermore, thinner absorb layers reduce the distance charge carriers must travel to reach electrodes, which decreases the possibility of recombination before collection. Thus, FF increases.

As shown in Fig. 10d, the efficiency conversion is enhanced since 29.96 to 32.30% at CuBi_2_O_4_ thickness growing since 0.65 µm to 3 µm, while the band gap of CuBi_2_O_4_ decreasing from 1.50 to 1.3 eV. The η value decreases from 29.96% to 13.55% when the band gap is shifted to higher values (higher than 1.50 eV), and layer absorber thickness is shifted to lower thickness values (lower than 0.65 µm). In general, higher efficiency values are generally observed at lower band gaps of CuBi_2_O_4_ and higher thicknesses. It is proposed that a layer absorber thicker and a smaller band gap will significantly boost photocurrent, improving the SCs overall performance^76^.

#### Variations in donor concentration as a result of CuBi2O4 thickness

**Fig. 11:**
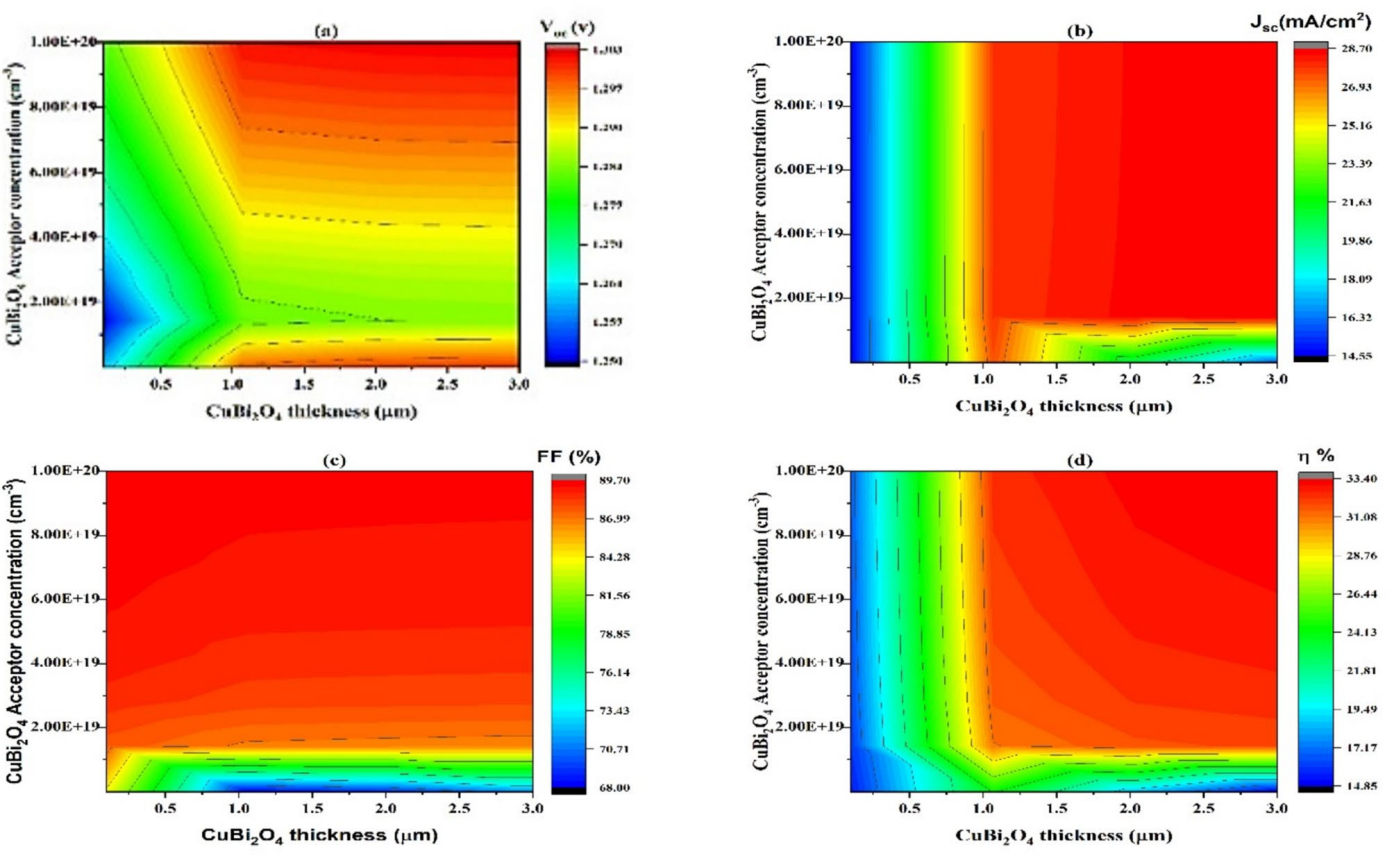
Important characteristics of the modeled SCs, displaying the open-circuit voltage (V_oc_) (**a**), current density of short-circuit (J_sc_) (**b**), fill factor (FF) (**c**), and (**d**) efficiency (η) in relation to the absorber thickness of the layer (x-axis) and concentration acceptor (y-axis).

A contour plot is shown in Fig. 11, to explain the relationship between three variables: CuBi_2_O_5_ thickness, this varies from 0.5 to 3.0 µm on the horizontal axis.; CuBi_2_O_4_ acceptor concentration, displayed on the vertical axis, ranging from $0.5\times$ ${10}^{18}$ to $1\times$ ${10}^{20}$ cm^-3^, and J_sc_, V_oc_, FF and values of efficiency, which are expressed by the color gradient.

According to Fig. 11a, voltage of open-circuit (V_oc_) values range since 1.250 to 1.303 V. Also, the values of open-circuit voltage (V_oc_) show several trends. Firstly, the value of V_oc_ is between 1.250 V and 1.290 when the thickness of CuBi_2_O_4_ is less than 0.7 µm, while CuBi_2_O_4_ acceptor concentration increases. Secondly, at a lower acceptor concentration below $1\times$ ${10}^{19}$ cm^-3^, and with increasing the thickness of CuBi_2_O_4_ above 0.7 µm, there is an increase in the value of V_oc_ from approximately 1.290 V to 1.303 V. Thirdly, there is a decrease in V_oc_ values at the zone of acceptor concentration ranging between $1\times$ ${10}^{19}$ cm^-3^ and $4\times$ ${10}^{19}$ cm^-3^, while CuBi_2_O_4_ thickness increases. Finally, higher V_oc_ values are generally observed at higher CuBi_2_O_4_ thickness and acceptor concentrations^77^.

As seen in Fig. 11b, the current density of short-circuit rises roughly as much as 28.7 mA/cm^2^ as the thickness raises from 1.0 to 3 µm and the concentration of acceptors in the absorb layer increases over 1 $\times$ ${10}^{19}$ cm^-3^. Also, lowest J_sc_ values are found among 14.55 mA/cm^2^ then 17.7 mA/cm^2^, where CuBi_2_O_4_ thickness is lower than 1 µm. The observed slight reduction in J_sc_ at very high doping levels can be attributed to increased recombination rates and reduced carrier mobility due to impurity scattering^78^.

In Fig. 11c, increased FF leads to increased acceptor concentrations, reaching approximately 86.99–89.7% within the CuBi_2_O_4_ acceptor concentration range of $1.5\times$ ${10}^{19}$–$1\times$ ${10}^{20}$ cm^-3^. For an acceptor concentration lower than $1.5\times$ ${10}^{19}$ cm^-3^, the FF is notably lower between 68.00 and 86.99%.

Figure 11d shows the conversion efficiency η is raised from 17.70 to 33.40% with the thickness of CuBi_2_O_4_ augmented since 0.1 µm near 3 µm, while acceptor concentration of CuBi_2_O_4_ increased from $1.5\times$ ${10}^{19}$ to $1\times$ ${10}^{20}$ cm-3. The η value decreases from 17.70% to 14.85% when the thickness is shifted to lower values (lower than 0.1 µm) while the acceptor concentration doesn’t affect, and the absorber layer of acceptor concentration is shifted to lower acceptor concentration values (lower than $1.5\times$ ${10}^{19}$ cm^-3^) while the thickness doesn’t affect. In general, higher efficiency values are generally observed at higher acceptor concentrations of CuBi_2_O_4_ and higher thickness.

#### Impact of CuBi2O4 thickness on J-V curve

**Fig. 12:**
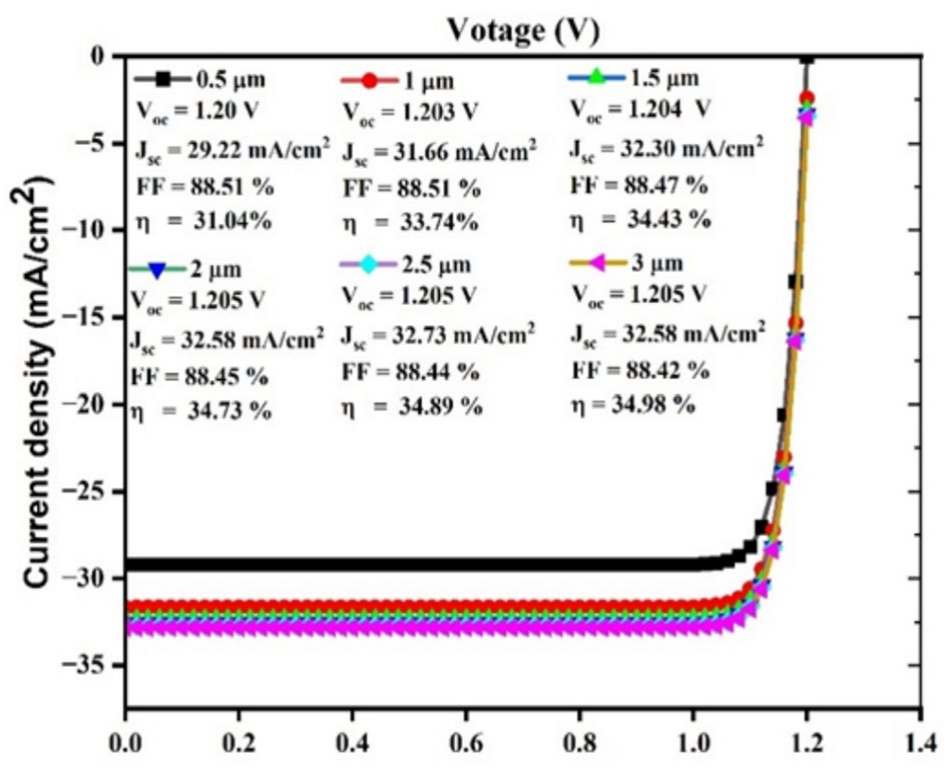
The J-V characteristic curve of the proposed SC by adjusting the thickness of the absorber layer.

The J-V features occur illustrated in Fig. 12, by adjusting absorber layer’s CuBi_2_O_4_ thickness. Here, layer of buffer, HTL, and ETL settings remain stable, while the absorber layers of CuBi_2_O_4_ varies from 0.5 µm to 3 µm. It can be found that current density and voltage increase with a continuous rise efficiency from 31.04% to 34.98% as the absorber thickness increases. As the absorb layer thickness increases, electrons and holes are added, increasing voltage and current density. This curve provides insight into how the SC is optimized for performance. It is ideal that the thickness of the absorbent layer be between 2.5 µm and 3.0 µm.

#### Impact of CuBi2O4 thickness, carrier concentration, and band gap on EQE

**Fig. 13:**
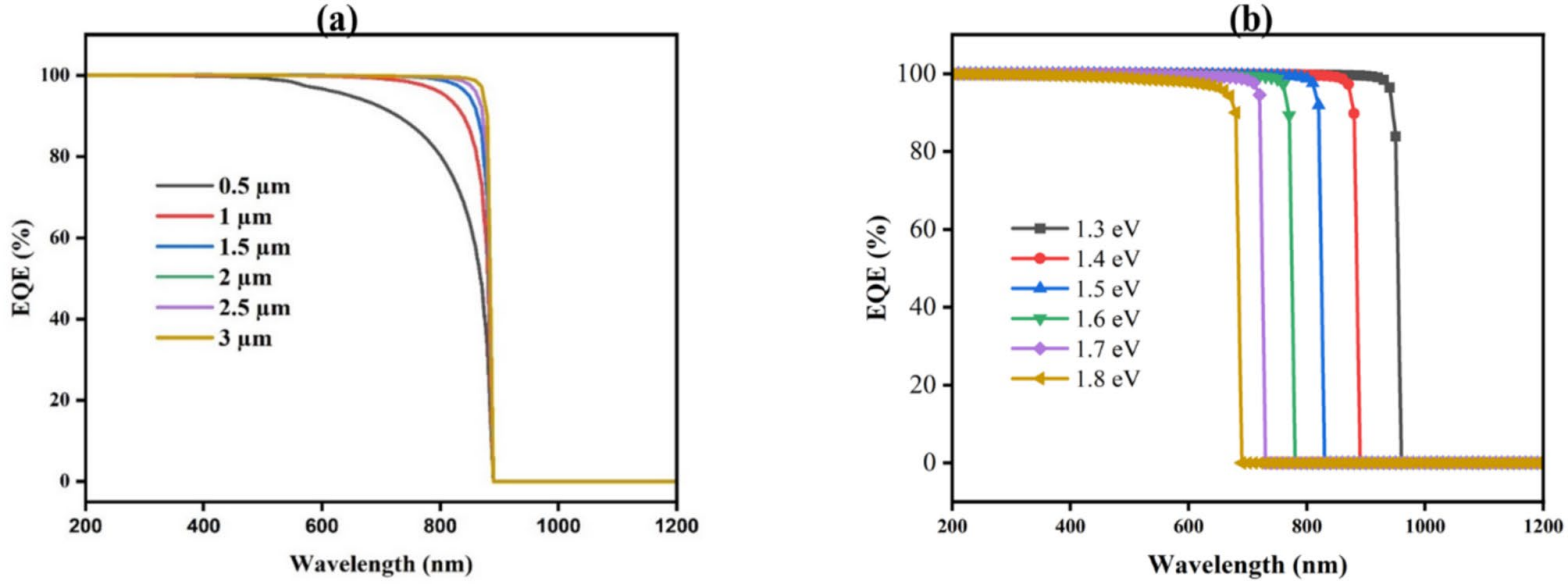
The wavelength dependent on quantum efficiency for different absorber (**a**) thickness, and (**b**) band gap.

Based on Fig. 13, the EQE of SCs with different thicknesses of CuBi_2_O_4_ absorb layer as a function of wavelength has been plotted as a function of wavelength. PV devices are measured by how many charge carriers they can extract from incident photons (shown in Fig. 13a)^79^.

The wavelength range of 200 to 1200 is used to evaluate the EQEs. A thickness of 0.5 to 3 µm is typical for the absorber layer. Figure 13 shows that the EQE of CuBi_2_O_4_ growths at extensive wavelengths as rise of thickness of layer absorber increases. For wavelengths less than 800 nm, all curves show strong EQE values (almost 100%). Throughout their thickness, SCs convert shorter-wavelength (higher-energy) photons into charge carriers efficiently. Moreover, CuBi₂O₄'s intrinsic absorption properties dominate the range below 800 wavelengths. As a result, fewer electron–hole pairs are generated by photons within the absorb layer.

Figure 13b illustrates the EQE with varying band gaps. The horizontal axis shows the wavelength of the incoming light, and the vertical axis exhibits the EQE. It’s noticed that at low band gaps (1.3–1.4 eV), a high EQE up to longer wavelengths (~ 950–1000 nm) is due to the ability to absorb low energetic photons, which result in effective carrier generation. At wavelengths of 800–900 nm, the band gaps of 1.5–1.6 eV also show high EQE. Higher band gaps (1.7–1.8 eV) are limited to absorbing higher-energy photons (shorter wavelengths), leading to a sharp drop in EQE beyond the band gap energy. So, for a broader spectrum of photons, low band gap material is needed to extend the EQE to a high value. However, this can decrease V_oc_ and FF due to higher recombination rates. Hence, balanced energy gaps (1.5–1.6 eV) are required for an overall optimized structure^80^.

### Defect density at the interface affects cell performance

**Fig. 14:**
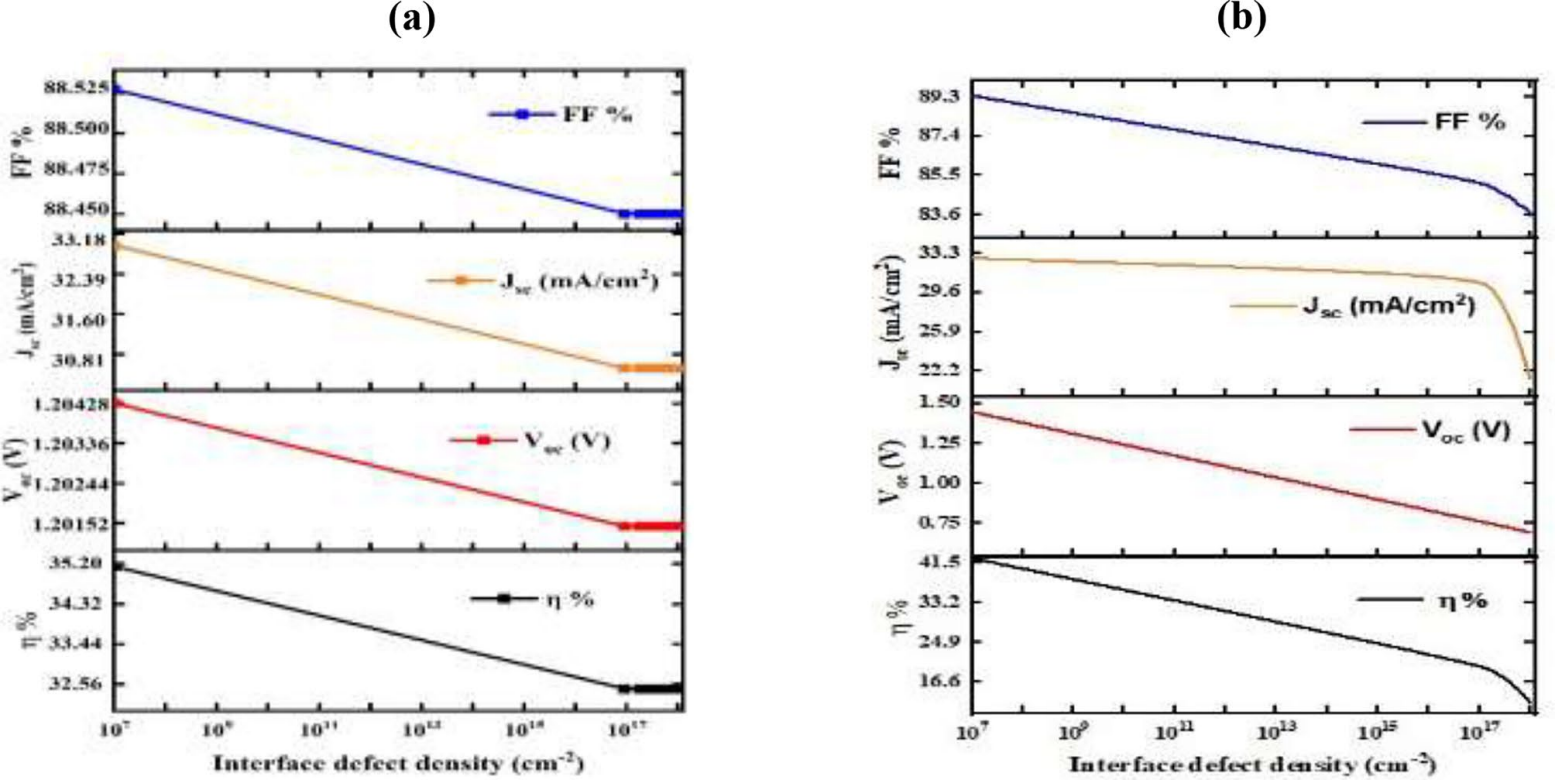
Influence of weakness density on η, V_oc_, J_sc_ and FF of cell at (**a**) Cu_2_O/CuBi_2_O_4_ and (**b**) CuBi_2_O_4_/CdS interfaces.

Defect density remains one of the most influential factors in determining the actual performance of thin-film solar cells. It’s observed strong correlations between lower defect levels and improved carrier lifetimes, reduced recombination, and overall higher efficiency. Deficiencies are at the interface between the HTL and the absorber as well as the buffer and the absorber. According to (Fig. 14), is analyzed in this numerical study in terms of cell performance. This Fig exhibits the impact of increasing defect density from 10^7^ to 10^18^ cm^-2^ at the Cu_2_O/CuBi_2_O_4_ and CuBi_2_O_4_/CdS interfaces on η, J_sc_, V_oc_, and FF cell. It is known that interface defects can reduce the transport of carriers and increase recombination, thus reducing cell performance^81^. As indicated by Fig. 14a, the result of increased density weakness at interface of Cu_2_O/CuBi_2_O_4_ on η, , J_sc_ ,V_oc_ and FF. It is noted that when the density weakness interface rises since 10^7^ near 10^17^ cm^-2^, the η, V_oc_, J_sc_, and FF decrease from 35.2 to 32.46%, from 1.20428 to 1.20145 V, from 33 to 30.5 mA/cm^2^, and from 88.525 to 88.45%, respectively. The values ​​remain constant after 10^17^ cm^-2^. As it is clear from (Fig. 14b), the effect of higher density weakness at interface of CuBi_2_O_4_/CdS on η, V_oc_, J_sc_, and FF. Which shows that the η and FF reduce non-linearly from 42.3 to 12.2% and from 89.3 to 83.5%, respectively, upon increasing of interface defect density from 10^7^ to 10^18^ cm^-2^. The V_oc_ decreases linearly from 1.45 to 0.7 V when the density weakness interface rises from 10^7^ to 10^18^ cm^-2^. The J_sc_ declined slightly from 32.8 to 30.34 mA/cm^2^ with a rise in weakness density interface since 10^7^ to 10^17^ cm^-2^ was significantly reduced since 30.34 to 21.4 mA/cm^2^ with a rise in weakness density interface since 10^17^ to 10^18^ cm^-2^. We can emphasize the relevance of microstructural engineering strategies such as surface passivation and grain boundary modification. These techniques play a critical role in reducing non-radiative recombination and enhancing the electrical properties of the absorber material. By minimizing defect-related losses, these measures directly contribute to improved device stability and performance^82^.

### Effects of ETL on structure of the SCs

The Electron Transport Layer (ETL) is integrated into the SCs structure because it makes it easier to remove electrons from the layer of activity and direct those electrons towards the electrode^83^. The outcomes are quite encouraging; however, losses still occur because of inappropriate band structure, carrier recombination, low carrier collecting efficiency, insufficient absorption, etc.; therefore, improving the device’s performance is probably inevitable. Furthermore, shunting among opaque conductive oxide and the absorber layer can be avoided by using the ETL^84^. At the absorber/ETL interface, generally speaking, there are two types of energy band structures: spike-like (E_C(Etl)_ > E_C(absorbance)_) and cliff-like (E_C(Etl)_ < E_C(abs.)_)^85^.

The primary function of ETL is to prevent holes from developing, collect electrons as of layer absorber, and transmit them to the anode. However, the development of ETL, as well as its relationship with low temperatures and efficient charge transport capabilities, is critical.

#### Effect of TiO_2_ thickness on variation of band gap

**Fig. 15:**
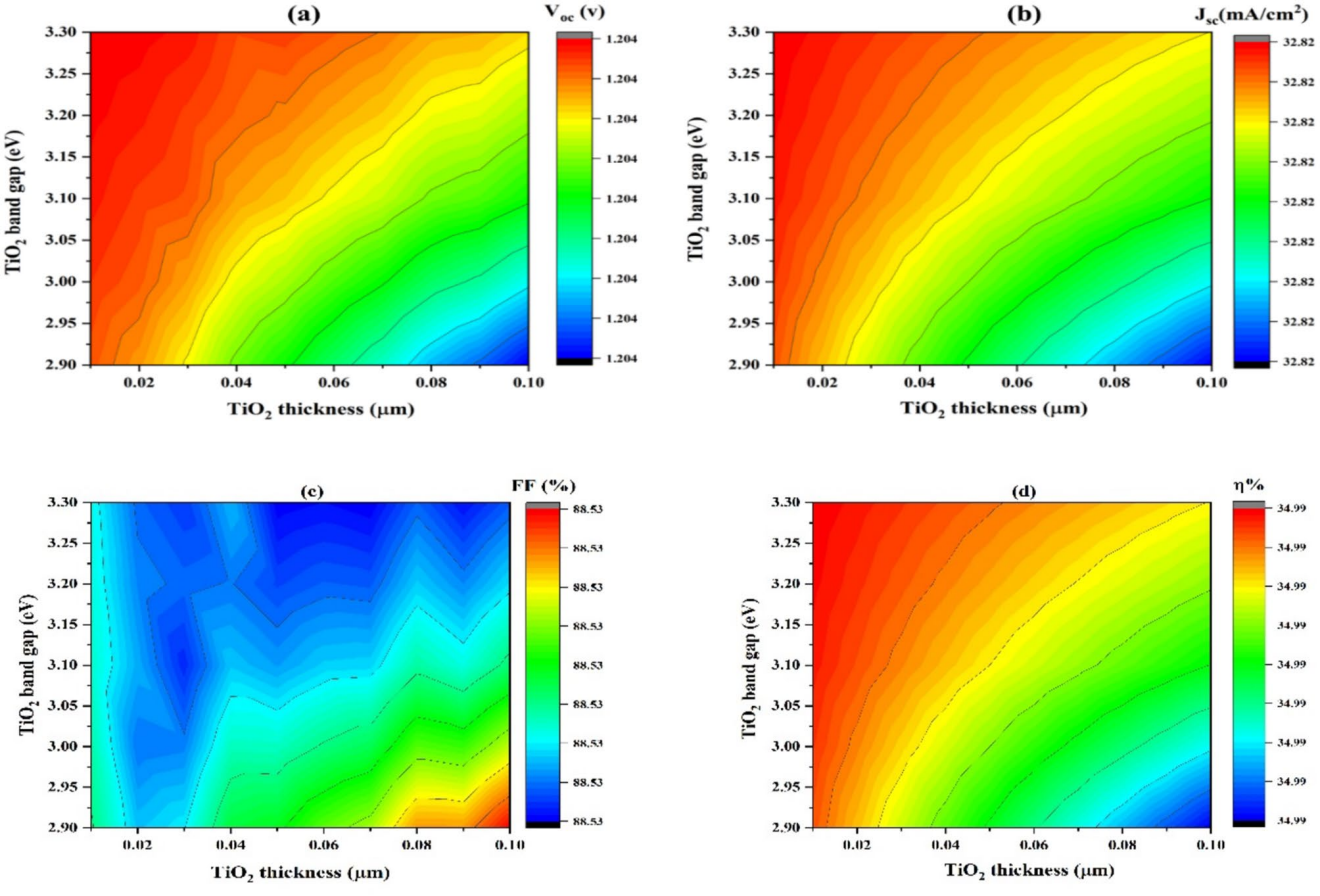
Influences of the (x-axis) thickness and (y-axis) band gap of TiO_2_ on (**a**) V_oc_, (**b**) J_sc_, (**c**) FF, and (**d**) η.

Changing the ETL’s thickness in the SCs can significantly enhance its performance^86^. To elevate the HOMO significantly higher than the absorber layer, the LUMO must be lower, and ETL must establish band alignment across the absorber substrate. Convolutional contour plots are displayed in (Fig. 15), illustrating the correlations between TiO_2_ band gap energy (eV), TiO_2_ thickness (µm), and parameters relating to photovoltaics: (J_sc_), (η), (V_oc_), and (FF). There is a range of 2.90 eV to 3.30 eV for the TiO_2_ band gap and 0.02 to 0.10 µm for TiO_2_thickness.

Furthermore, modifying the bandgap of SCs materials is crucial. SCs efficiency and overall performance are significantly affected by a material’s bandgap, which describes its electrical and optical features. The band gap configuration at the absorber and ETL interface determines the energy activation (E_a_) interface recombination of carriers. The recombination at interface decreases with increasing E_a_^87,88^.

With a decreasing band gap and increasing thickness, a lower open-circuit voltage (V_oc_) is observed in Fig. 15a, contour plot displays descending values (green, blue) and increasing values (red, yellow) for larger band gaps (3.20 eV to 3.30 eV) and thinner TiO_2_ layers (0.02 µm to 0.04 µm). However, V_oc_ exhibits a constant value of 1.204 V for all TiO_2_ band gaps and thickness changes. For higher V_oc_, the TiO₂ conduction band should align well with the donor’s LUMO. The consistency of the plot suggests that, within the range under investigation, variations in these parameters have no effect on the V_oc_, which may be because of the basic characteristics of the materials utilized, the V_oc_'s inherent limits in the specific material structure and configuration of devices, or the measurements could be carried out in well-controlled settings to provide reliable V_oc_ results.

The short-circuit current density (J_sc_) plot displays greater values for thinner TiO_2_ layers and higher band gaps, which can be expressed in (Fig. 15b), while decreasing values as thickness and band gap increase. Beyond 0.04 µm thickness and below 3.10 eV band gap, J_sc_ decreases, respectively, considering that charge carriers must travel a longer distance as thickness rises, increasing recombination. On the other hand, lower band gaps provide charge carriers with less energy, which decreases their ability to make contributions to the current. The J_sc_ value attained, 2.82 mA/cm^2^, is the utmost that can be achieved in the circumstances. Thus, this optimum value is unaffected by changes in band gap and thickness. Nonetheless, there may be several explanations for J_sc_'s consistency. One of them is that the apparatus might have reached a saturation point as recombination losses are reduced to the point that further band gap increases or thickness reductions will not increase^89^. Within the tested parameter space, the device appears to function dependably, as indicated by the constant J_sc_ in our study’s region.

Figure 15c, illustrates the constancy of Fill Factor (FF) at the value of 88.53% when the TiO_2_ thickness was wide-ranging since 0.02 to 0.10 µm also, energy gap at 2.90 eV to 3.30 eV, indicating that the majority of the plot shows a stable FF value regardless of the variations in thickness and band gap. At a thickness higher than 0.06 µm and a band gap less than 3.10 eV, FF decreases slightly from 88.53% to 88.50%, indicating that the material properties or device conditions might be less optimal, leading to slightly higher recombination rates or increased resistance. whereas at thickness within sort of 0.02 to 0.06 µm and an energy gap ranging from 3.10 eV to 3.30 eV, FF slightly increases back to the constant value of 88.53%. This indicates areas where the material properties or device conditions are improving, reducing recombination rates. The manufacturing and SCs design are tuned to keep the FF high. A stable FF is the result of low recombination losses and good charge collection efficiency, which are ensured by elements including the contacts, interfaces, and overall device architecture.

According to Fig. 15d, the illustration of the efficiency (η) contour plot shows the SCs difference in efficiency through TiO_2_ band gap (eV) and thickness (µm). A measure of efficiency 34.99% was opined by sorts of thickness 0.02 to 0.04 µm and band gaps of 3.20 to 3.30 eV. The layer’s overall efficiency is unaffected by the minor variations in the energy gap and thickness. The interactions between the TiO_2_ band gap and thickness, which have an impact on the charge carriers’ dynamics and recombination rates, are what cause the shifts in the plot.

Nevertheless, the alignment of acceptor and donor materials’ HOMO and LUMO levels with TiO_2_ band structure is necessary for the solar device to operate at its best. Thus, the provided thicknesses and band gap ranges are provided for better alignment, leading to higher efficiency.

#### Influences of changing the donor carrier concentrations of TiO_2_

Higher donor carrier concentrations increase the electrical conductivity of TiO_2_. Increasing donor carrier concentration brings the level of Fermi nearer to the conduction band. This change may enhance the band’s coherence between the TiO_2_ ETL and the neighbouring layers. Therefore, adjusting the donor carrier concentration in TiO_2_ can have a significant impact on the device’s efficiency^90^.

**Fig. 16:**
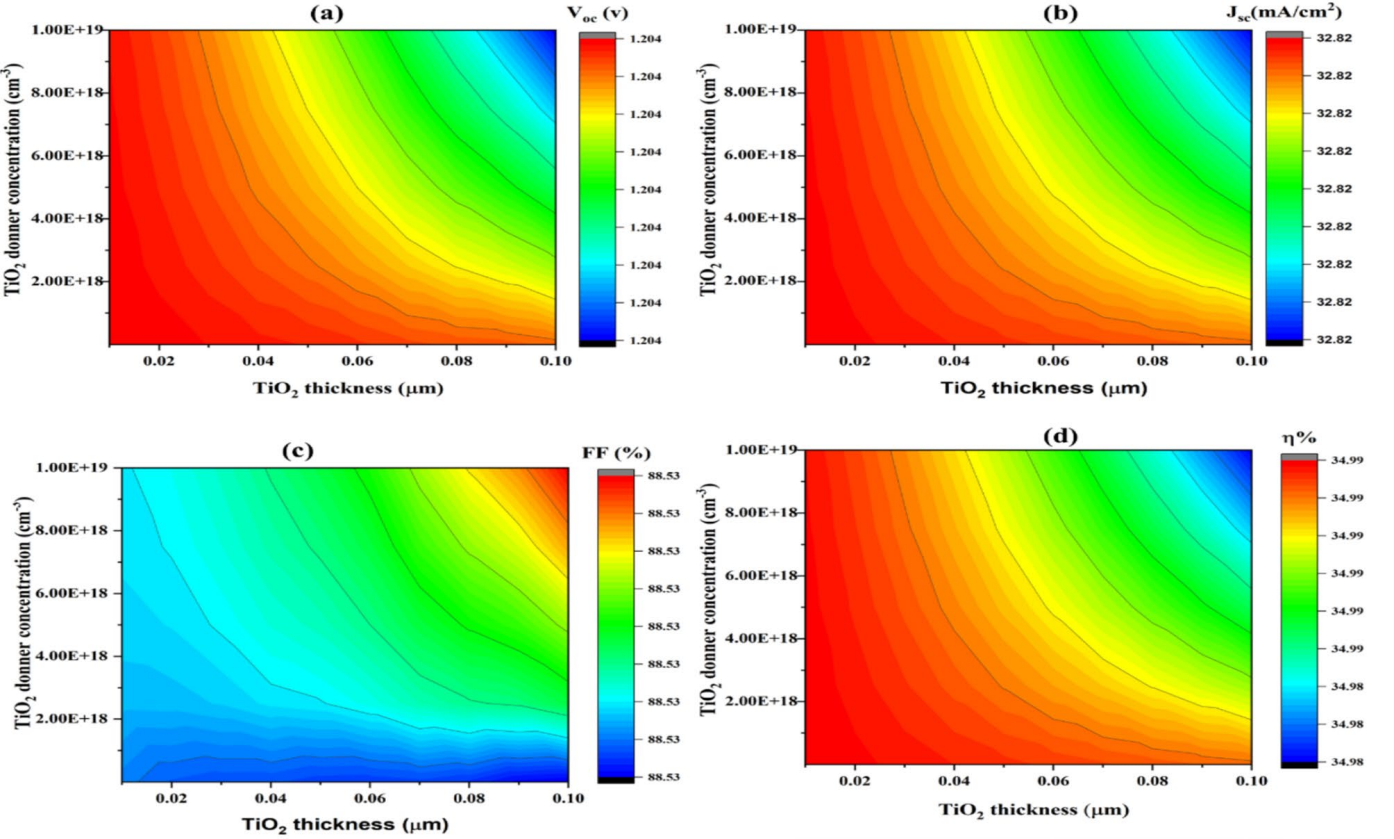
Represents the parameters of a simulated SCs, with TiO_2_ thickness and carrier concentration, owning an unchanging energy band gap of 3.25 eV. The parameters are (**a**) Voc, (**b**) Jsc, (**c**) FF, and (**d**) η.

The acquired photovoltaic primary parameters V_oc_, FF, J_sc_, and η are plotted as a contour plot (expressed in Fig. 16) against the TiO_2_ thin layer thickness on the x-axis (from 0.01 µm to 0.1 µm) and the carrier concentration (since 1 × 10^18^ to 1 × 10^19^ cm⁻^3^) on the y-axis in order to examine how different TiO₂ thicknesses and donor concentrations impact the performance parameters.

As stated in Fig. 16a, V_oc_ remains constant at 1.204 V for every variation of TiO_2_ thickness (0 to 0.10 µm) and TiO_2_ donor concentration (1 × 10^18^ to 1 × 10^19^ cm⁻^3^). This constancy indicates that the V_oc_ is steady and not greatly altered within the specified range of these parameters. This stability in V_oc_ is dependent on the layout of structure SCs.

Figure 16b, clearly displays that J_sc_, which represents the highest current the cell can produce under illumination without any external load, is constant at 32.82 mA/cm^2^ for all values of TiO_2_ thickness and donor concentration. The color bar shows that J_sc_ does not vary because the plot is entirely identical in color. Regardless of variations in the TiO_2_ layer characteristics within the specified range, this suggests that the SCs retain a high degree of efficiency (in terms of current generation). This suggests that the materials and design have been carefully considered to ensure smooth operation.

While in Fig. 16c, the fill factor (FF) graph, which ranges from 88.53% to 88.83%, is high and stable, indicating that changes in donor concentration and TiO_2_ thickness have little to no impact. This illustrates the endurance and high efficiency of SCs design, along with its minimal losses and efficient charge carrier collection.

In addition, Fig. 16d shows that the efficiency (η) remains constant at 34.99% despite changes in donor concentration and TiO_2_ thickness. Since the entire plot is colored the same, the color bar shows no variance. The efficiency consistency reflects the functionality of the SC, which is highly stable. Specifically, thickness and TiO_2_ doping altitudes layer are probably in an ideal range that does not adversely affect performance, and it is probably working effectively as a charge transport layer.

#### Modeling of the J-V curve

**Fig. 17:**
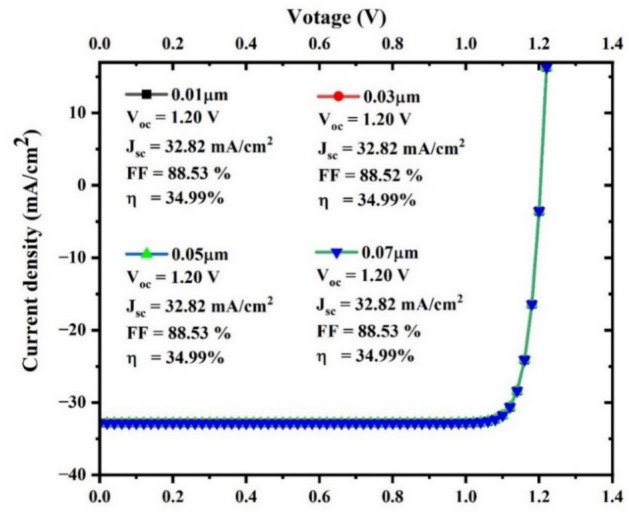
J-V curve analysis.

The J-V curve, which represents the amount of absorbed sunlight converted to electrical energy, is an important performance metric. It was conducted as part of our research, which is illustrated in (Fig. 17).

To determine the effectiveness of the structure of the SC, the current density–voltage (J-V) curve was plotted between current density (y-axis) and voltage (x-axis), incorporating variations in TiO_2_ thickness (0.01, 0.03, 0.05, and 0.07 µm). The graph effectively demonstrated that all the curves have identical values for V_oc_, J_sc_, FF, and η for all thicknesses. The uniform charge carrier generation and collection, which are related to material thickness, may be the reason for the parameter constancy. If the material’s absorption depth is less than the thinnest film (0.01µm), all of the films would absorb the same amount of light and generate a similar number of charge carriers despite their thickness. Also, the thickness range (0.01µm to 0.07µm) is quite narrow. The effect of thickness change on device performance may be negligible within this narrow range, particularly if the active layer is already tuned for effective charge transport and light absorption. This suggests that the variations in thickness do not affect charge transportation, recombination behaviour, or collection productivity. However, each curve has an identical shape and sharply rises in the vicinity of the open-circuit voltage, signifying high diode performance and efficient charge collection. As determined by the fill factor, 88.52 to 888.53%, the device has a high shunt resistance and a low series resistance. FF and η have the highest values at the ideal thickness of 0.05 µm, while V_oc_ has the lowest value. At 0.05µm, the film is thin enough to minimize material usage but thick enough to ensure uniform deposition and handling stability.

#### Effect of the TiO_2_ EQE

**Fig. 18:**
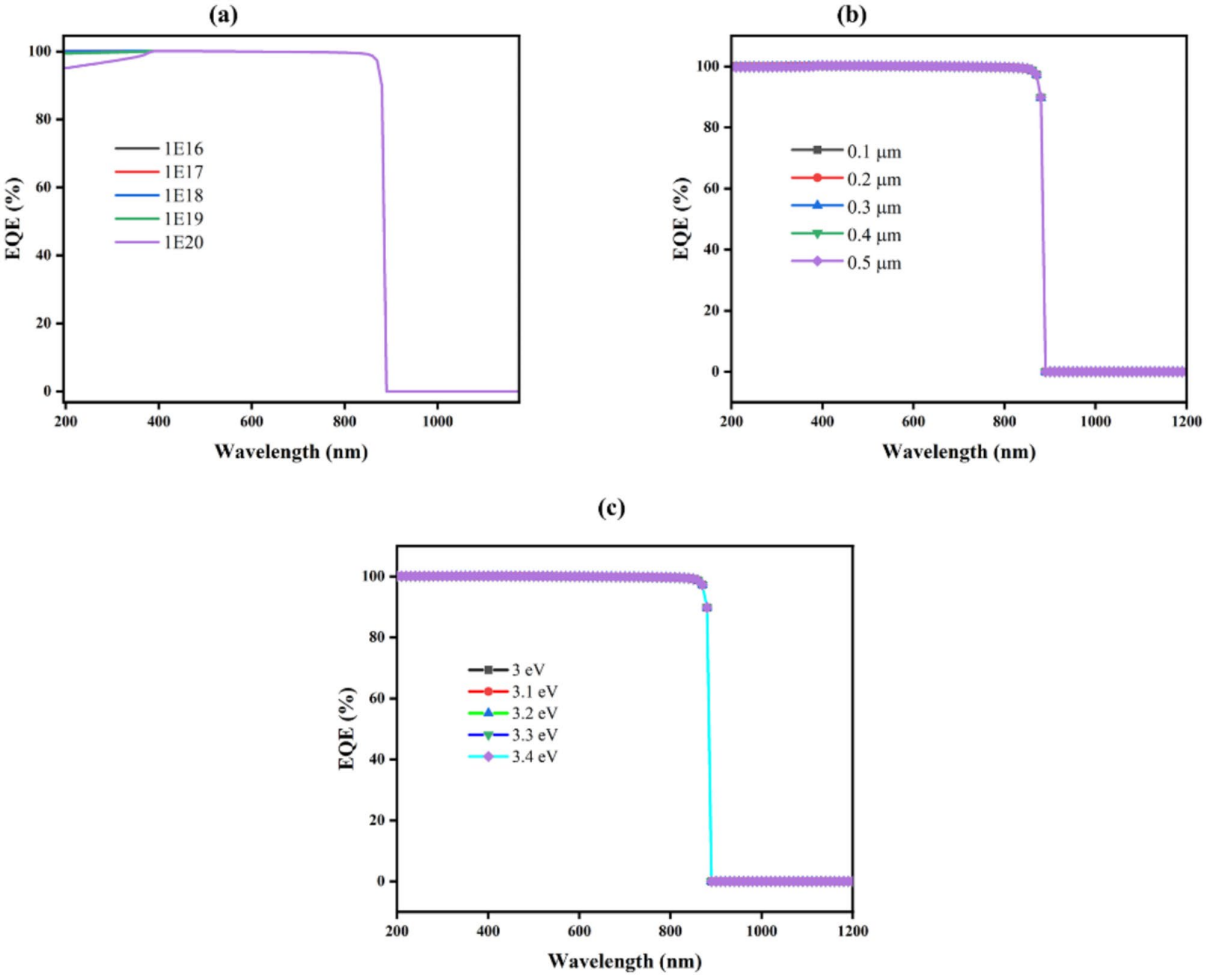
The wavelength-dependent quantum efficiency for various ETL (**a**) thickness, (**b**) donor concentration, and (**c**) band gap.

EQE illustrates how sensitive a SC is to changed elements of spectrum of solar, expressed as a wavelength function. Additionally, the fraction of light photons transformed into charge carriers is measured by EQE^91^. The EQE curve can be used to identify the precise wavelengths at which the SCs function. Considering different conditions such as thickness, donor concentration, and band gaps are all being noticed. Figure 18 shows the EQE spectra at wavelengths between 200 and 800 nm.

Figure 18a depicts the variation across different levels of doping (10E^16^ to 10E^20^ cm^-3^) for the SCs EQE spectrum, indicating that in the visible range (400–800 nm), the graph shows a high EQE near 100%, which suggests effective photon-to-electron conversion over a broad range of doping concentrations. However, beyond about 800 nm, which is the material’s bandgap energy, there is a significant reduction in EQE, indicating that photons with longer wavelengths are not efficiently absorbed.

While in Fig. 18b, the EQE spectra for SCs are illustrate with increasing in the active layer thicknesses (0.1 to 5 µm). With EQE values in the 400–800 nm range that are nearly identical and close to 100%, all thicknesses exhibit effective charge collection. The drop in EQE beyond 800 nm remains consistent across different thicknesses, highlighting that the absorption edge is primarily figured through material’s intrinsic characteristics instead of thickness.

The illustration of EQE spectra for SCs with different bandgap energies (3 eV to 3.4 eV) can be noticed, respectively, in (Fig. 18c). Bandgap energy influences a rapid reduction in EQE at longer wavelengths; greater bandgap energies show a drop at shorter wavelengths, showing EQE of nearly 100% across the visible spectrum (400–800 nm).

Generally speaking, the thickness, donor concentration, and band gap modifications all have barely any impact on the EQE.

### Effect of replace gold (Au) as a back contact with molybdenum (Mo)

**Fig. 19:**
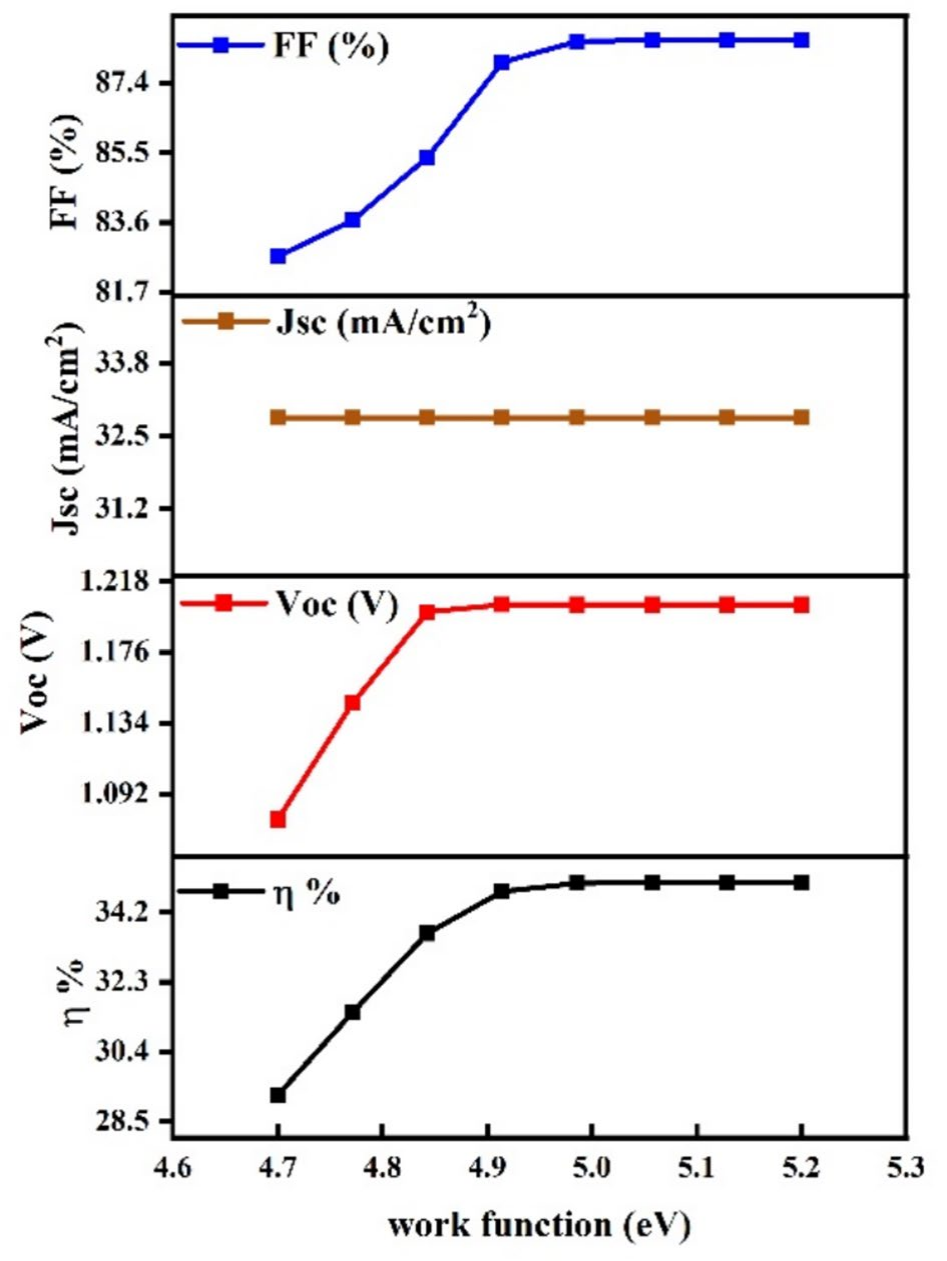
Impact of Molybdenum (Mo) Back contact on solar cell efficiency and key performance parameters.

This graph illustrates the performance of a solar cell using Molybdenum (Mo) as the back contact instead of Gold (Au). Although Mo is more environmentally friendly and cost-effective, the overall efficiency decreases. As shown, the fill factor (FF) improves with increasing work function, reaching about 85.5%, while the short-circuit current density (J_sc_) remains nearly constant at 33.8 mA/cm^2^. The open-circuit voltage (V_oc_) also rises and stabilizes near 134 V. However, despite these factors, the efficiency (η) is lower compared to Au, reaching just above 32.2%. as shown in (Fig. 19). This indicates that replacing Au with Mo leads to a reduction in solar cell efficiency despite its environmental benefits.

### Impact of defect density on the CuO/CuBi_2_O_4_ and CuBi_2_O_4_/CdS interfaces on solar cell performance

**Fig. 20:**
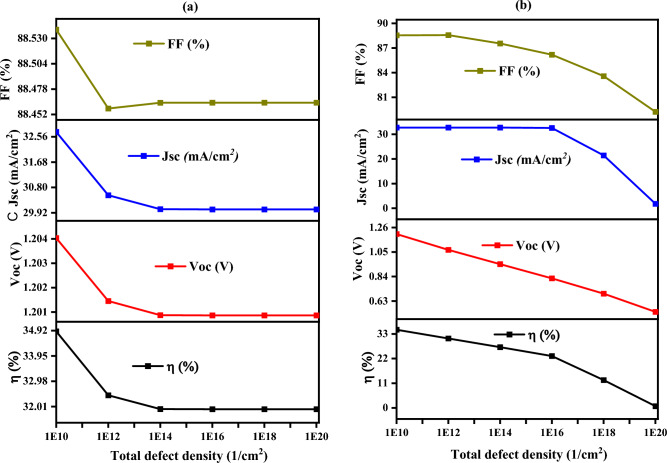
Impact of defect density on solar cell performance at the interfaces between (**a**) CuBi_2_O_4_/CuO and (b) CuBi_2_O_4_/CdS.

In Fig. 20a, as defect density increases, the fill factor (FF) remains mostly stable, with minimal impact on charge collection efficiency. However, short-circuit current density (J_sc_) decreases from 33 mA/cm^2^ to 30 mA/cm^2^, highlighting increased recombination at the interface. The open-circuit voltage (V_oc_) shows a slight decline from 1.204 V to 1.201 V, suggesting minor effects on the voltage generated by the solar cell. Overall efficiency (η) decreases from 35.2% to 32.5% due to the increased recombination, which reduces the cell’s ability to convert light into electricity^92^. In Fig. 20b, at this interface, the fill factor (FF) decreases significantly from 89 to 85% as defect density increases, indicating a more substantial impact on charge collection efficiency compared to the CuO/ CuBi_2_O_4_ interface. The short-circuit current density (J_sc_) drops from 32.8 mA/cm^2^ to 29.5 mA/cm^2^, and the open-circuit voltage (V_oc_) experiences a sharp decline from 1.20 V to 1.10 V due to higher recombination at this interface. The efficiency (η) also decreases substantially from 42.3% to 33.2%, reflecting the severe performance degradation caused by defects at the CuBi_2_O_4_/CdS interface^93^.

### An ideal Cu_2_O/CuBi_2_O_4_/CdS/TiO_2_ solar cell and its JV curve at different operating temperatures

**Fig. 21:**
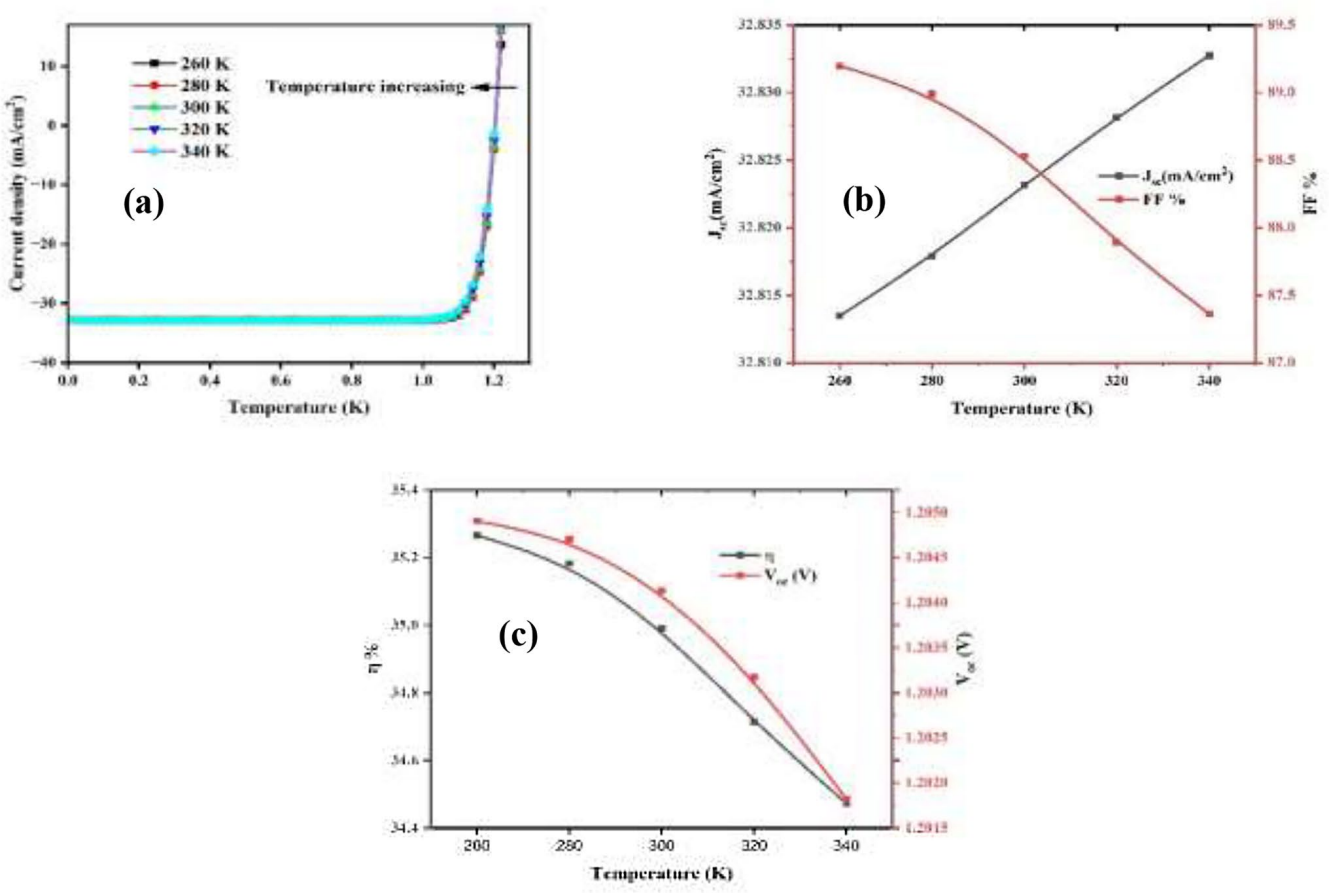
Effect of temperature of CuBi_2_O_4_ absorber layer on (a) current density (b) J_sc_, and FF and (c) V_oc_, and η.

Temperature has greatly affected the performance of the PSCs. As a result of overheating, performance fluctuation is a common occurrence^94^. It was found that with the increasing temperature, PCE, V_oc_, and FF of the solar cell decreased for both configurations, because the carrier concentrations, mobility of the charge carriers, resistance and bandgap of the materials alters at a higher temperature.^95^. Figure 21 illustrates the effect of increasing temperature from 260 − 340 K in regard to the absorbency layer’s parameters, namely, V_oc_, FF, J_sc_, and η. As shown in (Fig. 20a), increased J_sc_ values from -30 mA/cm^2^ to 10 mA/cm^2^ were observed with increasing temperature. As explained in (Fig. 21b), there was a small rise in J_sc_ values from 32.815 to 32.835 mA/cm^2^, with a decrease in the FF percentage. As a result of thermal agitation, charge carriers are extracted^96^. The dark saturation current and the load zone spatial size are directly related to n_i_^2^ concentration; getting higher the intrinsic concentration raises the saturation current density and decreases the open circuit voltage (V_oc_)^97^. The V_oc_ value decreased from 1.2047 V at 273 K to 1.2018 V at 340 K, with efficiencies of 35.25% at 273 K and 34.44% at 340 K, as shown in (Fig. 21c). The increase in temperature increases the likelihood of recombination among charge carriers before they reach the depletion region, lead to V_oc_ reduce in and FF^98^. Elevated temperatures decrease the semiconductors’ velocity stability, bandgap energy, and open-circuit voltage while increasing the reverse saturation current and resistivity. The temperature dependency of the I_o_ causes the V_oc_ to drop with temperature growing as shown in Eq. (3). Because Io is temperature-dependent, the V_oc_ initially reduced with rising temperature^99^.

3 $$I_{0} = qA\frac{{Dn_{i}^{2} }}{{LN_{D} }}$$

The behavior of a minority carrier is determined by its diffusivity D, diffusion length L, doping level N_D_, and intrinsic carrier concentration n_i_. Among listed factors, temperature had the greatest effect on concentration of intrinsic carrier (n_i_). Modifying I_o_ term in V_oc_ equation affects the equation’s temperature sensitivity as shown in Eq. (4).

4 $$V_{oc} = \frac{KT}{q}\ln \frac{{I_{sc} }}{{I_{0} }}$$

**Fig. 22:**
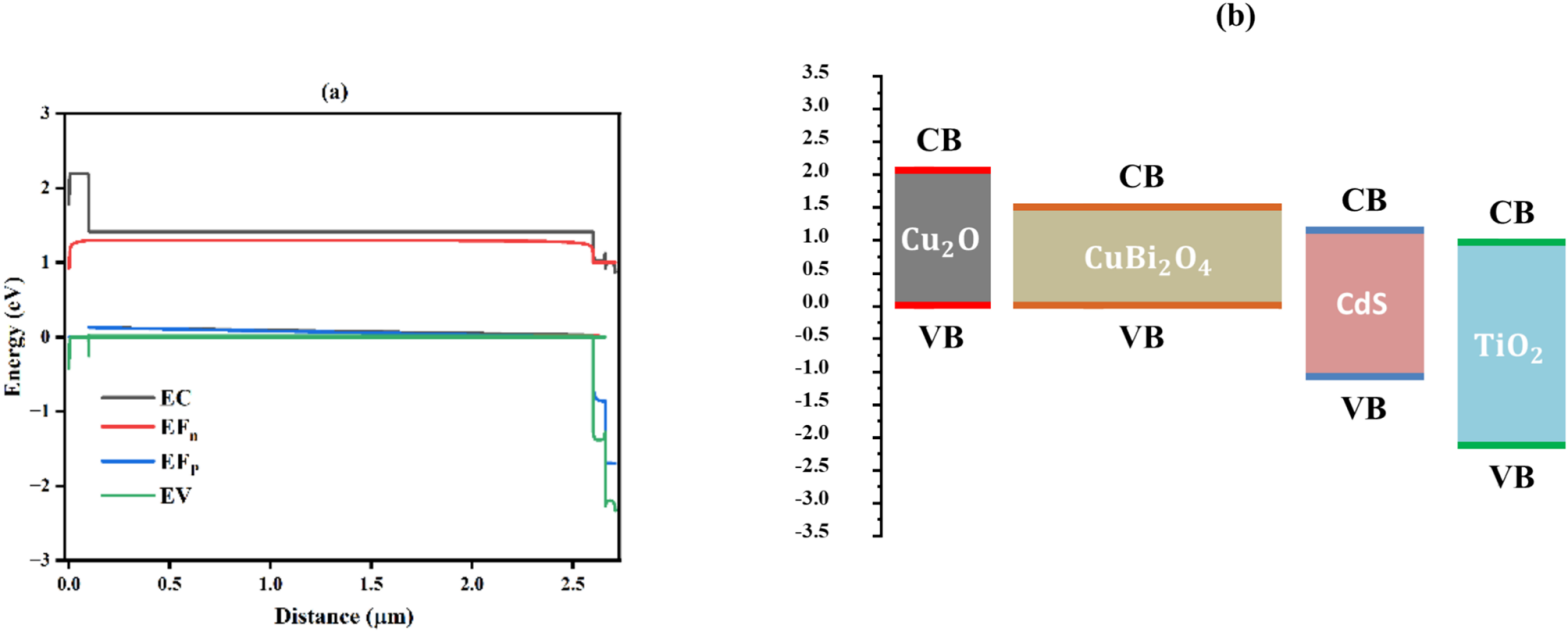
(**a**) Energy band diagram and carrier dynamics in a multilayer semiconductor device, (**b**) material stack and bandgap alignment in Cu₂O/CuBi₂O₄/CdS/TiO₂-based thin-film solar cell.

In Fig. 22a, the graph illustrates the energy band structure across a multilayer semiconductor device, showing the relationship between energy (in eV) and distance (in micrometers, μm). The conduction band energy (EC) starts at around 2 eV and decreases to 1 eV, reflecting a shift at the interface between materials with different electronic properties. The Fermi level for negative carriers (EF_n_) remains relatively constant at approximately 1.5 eV and then decreases to near 0 eV, indicating efficient electron transport. The Fermi level for positive carriers (EF_p_) initially stays near 0 eV before dropping sharply to -2 eV, suggesting regions of hole accumulation and depletion that help separate charges. The valence band energy (EV) starts at -1 eV and decreases further, indicating modifications due to material boundaries or doping. These energy band offsets, particularly at the material interface, guide charge carriers, suppress recombination, and enhance the efficiency of optoelectronic devices like solar cells^92^.

In Fig. 22b, the diagram illustrates the energy band structure of a multilayer semiconductor device with the material stack Au/Cu₂O/CuBi₂O₄/CdS/TiO₂/FTO, where each material has a distinct band gap: Cu₂O (2.1 eV) as the hole transport layer, CuBi₂O₄ (1.35 eV) as the absorber, CdS (2.3 eV) as the buffer layer, and TiO₂ (3.2 eV) as the electron transport layer. The band offsets between these materials are carefully aligned to facilitate efficient charge separation and transport. The conduction band of Cu₂O allows for hole extraction, while CuBi₂O₄ absorbs light and generates charge carriers, with its conduction band aligned to extract electrons into the CdS layer. CdS ensures efficient electron transport, and TiO₂, with its higher band gap, effectively blocks holes while allowing for electron movement, thus enhancing the overall efficiency of the device, particularly in applications like thin-film solar cells^93^.

## Optimization of CuBi₂O₄-based thin-film solar cell (TFSC)

This study evaluates the PV performance of CuBi_2_O_4_-based photovoltaic module. Table 4 shows the optimum physical characteristics for a highly efficient heterojunction Au/Cu_2_O/CuBi_2_O_4_/CdS/TiO_2_/FTO SC. At optimal physical parameters, the CuBi_2_O_4_ SC device achieves a maximum PCE of 32.56%, at V_oc_ of 1.27 V, J_sc_ of 32.83 mA/cm^2^, and FF of 88.7%. Optimizing these parameters can enhance overall device performance.

**Table 4:** Optimized CuBi₂O₄-based TFSC.

Optimized parameters (unit)	n-type ETL (TiO2)	n-type Buffer (CdS)	p-type Absorber (CuBi_2_O_4_)	p + -type HTL (Cu_2_O)	Interface defect density	
					CuBi_2_O_4_/CdS	Cu_2_O/CuBi_2_O_4_
Thickness (µm)	0.01	0.02	2.5–3.0	0.4		
N_D_ (cm^3^)	10^19^	10^16^	–	–		
N_A_ (cm^3^)	–	–	8 × 10^19^	10^19^		
σ_n,p_ (cm^2^)					10^–19^	10^–19^
Total density (cm^2^)					10^10^	10^10^

Table 5 Presents the simulated efficiencies, voltage, fill factors, and current densities for various device configurations. Notably, the study differentiates itself with a device structure constructed from Au/Cu_2_O/CuBi_2_O_4_/CdS/TiO_2_/FTO, which has impressive characteristics such as a V_oc_ of 1.2 V, a J_sc_ of 32.85 mA/cm^2^, an FF of 88.42%, as well as efficiency of 32.56%.

**Table 5:** Compares the results of the current study with different heterojunction CuBi_2_O_4_-based TFSCs.

Device structure	Field of research	Voc (V)	J_sc_ (mA/cm^2^)	FF (%)	η (%)	Reference
Au/CuBi_2_O_4_/SrSnO_3_/ITO	Theoretical	1.32	20.70	80.71	22.19	^100^
Au/CuBi_2_O_4_/WS_2_/ITO	Theoretical	1.33	21.08	81.3	22.84	^101^
Mo/CuBi_2_O_4_/CdS/FTO/Al	Theoretical	0.97	31.61	84.58	26.0	^46^
ITO/SnS_2_/CBO/Au		1.54	21.8	84.32	27.37	^101^
Mo/Cu_2_O/CuBi_2_O_4_/CdS/FTO/Al	Theoretical	1.02	32.49	87.91	29.2	^102^
Mo / CuBi_2_O_4_/TiO_2_/ITO/ Al	Theoretical	1.36	25.81	88.77	31.21	^103^
Ni/CuBi_2_O_4_/CdS/SnO_2_/Al	Theoretical	1.37	25.85	86.5	31.41	^104^
Ni/CuBi_2_O_4_/CdS/SnO_2_/Al	Theoretical	1.38	26.2	88.8	31.8	^105^
Au/ CuBi_2_O_4_/PCBM/ITO/Ag	Theoretical	1.32	27.46	88.69	32.16	^106^
Au/Cu_2_O/CuBi_2_O_4_/CdS/TiO_2_/FTO	Theoretical	1.20	32.58	88.42	32.56	This study

Evaluating these outcomes. to previous experiments. It is apparent that different device designs produce unique performance outcomes. This demonstrates the potential of Cu_2_O/CuBi_2_O_4_/CdS/TiO_2_ as a photovoltaic active layer, especially in terms of reaching evaluated performance among essential parameters.

## Conclusions

This study on CuBi₂O₄-based thin-film solar cells (TFSCs) shows how to enhance SC’s effectiveness through modelling and analysis using the SCAPS-1D program. The thickness of absorber layer, carrier concentration and band gap extensively affect the photovoltaic primary parameters, including current density of short circuit (J_sc_), voltage of open circuit (V_oc_), fill factor (FF), and efficiency (η). Adjusting these TFSC parameters optimally enhances overall efficiency.

- Using Cu_2_O as HTL increase efficiency to 30.89% with decreasing carrier concentration < 10^20^ cm^-3^ and increasing the thickness 0.05 to 0.4 µm and band gap less around 2.1 eV, which lead to a decrease in the recombination of photogenerated charges and enhanced electrical conductivity.
- Using CdS as buffer layer facilitate electron separation when the transfer band edge of CdS approaches or better aligns with the conduction band of CuBi_2_O_4_, resulting in a rise in the efficiency of SC.
- The alignment of the donor and acceptor materials’ HOMO and LUMO levels with the TiO_2_ (ETL) band structure is necessary for the solar device to operate at its best. Thus, the provided thickness, carrier concentration and band gap ranges are provided for better alignment, leading to higher efficiency
- Increasing the temperature from 260 − 340 K affects the absorber layer parameters by increasing the short-circuit current density (Jsc) slightly, while decreasing the open-circuit voltage (Voc), fill factor (FF), and overall efficiency (η) due to enhanced charge carrier recombination and thermal agitation.
- The device structure (Au/Cu_2_O/CuBi_2_O_4_/CdS/TiO_2_/FTO) achieved an optimized open-circuit voltage of 1.2 V, a short-circuit current density of 32.85 mA/cm^2^, a fill factor of 88.42%, and an efficiency of 32.56%, highlighting its potential in solar energy technology

This research contributes to the ongoing efforts to advance TFSC technology by comprehensively understanding the factors affecting CuBi₂O₄-based solar cell performance. The results lay a foundation for further experimental validation and the potential development of next-generation photovoltaic devices with improved efficiency and stability.

## Data Availability

The datasets used and/or analyzed during the current study available from the corresponding author on reasonable request.
